# Molecular Factors of Hypochlorite Tolerance in the Hypersaline Archaeon *Haloferax volcanii*

**DOI:** 10.3390/genes9110562

**Published:** 2018-11-20

**Authors:** Miguel Gomez, Whinkie Leung, Swathi Dantuluri, Alexander Pillai, Zyan Gani, Sungmin Hwang, Lana J. McMillan, Saija Kiljunen, Harri Savilahti, Julie A. Maupin-Furlow

**Affiliations:** 1 Department of Microbiology and Cell Science, Institute of Food and Agricultural Sciences, University of Florida, Gainesville, FL 32611, USA; migmez10@gmail.com (M.G.); whinkie1388@gmail.com (W.L.); swathidantuluri@ufl.edu (S.D.); pillai.alexander@gmail.com (A.P.); z.gani1997@ufl.edu (Z.G.); sungmin.hwang@duke.edu (S.H.); lana.mcmillan@locus-bio.com (L.J.M.); 2 Genetics Institute, University of Florida, Gainesville, FL 32611, USA; 3 Department of Bacteriology and Immunology, Immunobiology Research Program, University of Helsinki, 00014 Helsinki, Finland; saija.kiljunen@helsinki.fi; 4 Division of Genetics and Physiology, Department of Biology, University of Turku, 20014 Turku, Finland; harri.savilahti@utu.fi

**Keywords:** archaea, oxidative stress, hypochlorite, redox-active, proteasome

## Abstract

Halophilic archaea thrive in hypersaline conditions associated with desiccation, ultraviolet (UV) irradiation and redox active compounds, and thus are naturally tolerant to a variety of stresses. Here, we identified mutations that promote enhanced tolerance of halophilic archaea to redox-active compounds using *Haloferax volcanii* as a model organism. The strains were isolated from a library of random transposon mutants for growth on high doses of sodium hypochlorite (NaOCl), an agent that forms hypochlorous acid (HOCl) and other redox acid compounds common to aqueous environments of high concentrations of chloride. The transposon insertion site in each of twenty isolated clones was mapped using the following: (i) inverse nested two-step PCR (INT-PCR) and (ii) semi-random two-step PCR (ST-PCR). Genes that were found to be disrupted in hypertolerant strains were associated with lysine deacetylation, proteasomes, transporters, polyamine biosynthesis, electron transfer, and other cellular processes. Further analysis revealed a Δ*psmA1* (α1) markerless deletion strain that produces only the α2 and β proteins of 20S proteasomes was hypertolerant to hypochlorite stress compared with wild type, which produces α1, α2, and β proteins. The results of this study provide new insights into archaeal tolerance of redox active compounds such as hypochlorite.

## 1. Introduction

Reactive oxygen species (ROS) and other redox-active compounds can overwhelm the antioxidant mechanisms of a cell and cause damage to most biomolecules including proteins, nucleic acids, lipids, and carbohydrates [1,2]. Oxidation can lead to mutations in DNA by generating single- and double-stranded breaks in the backbone, crosslinks (interstrand and intrastrand), and adducts of bases and sugars [3]. Cell membrane lipids, when oxidized, lose flexibility, which can result in cell lysis [3,4]. Protein oxidation is particularly disruptive, as it leads to protein misfolding, aggregation, breaks in the protein backbone, modified amino acid residues, and loss of catalytic function causing bottlenecks in metabolism [5].

Haloarchaea thrive in hypersaline environments associated with high concentrations of chloride, high doses of ultraviolet (UV) irradiation, oxidative stress, osmotic stress, desiccation, and other extreme conditions [6]. Hypochlorous acid (HOCl) and its derivatives are redox-active compounds commonly encountered in environments of high concentrations of chloride [7,8]. In solution, sodium hypochlorite (NaOCl) forms sodium hydroxide (NaOH) and the strong oxidant HOCl, which can dissociate into hydroxide (OH^−^) and hypochlorite (OCl^−^) anions [2].

NaOCl + H_2_O ⇔ NaOH + HOCl ⇔ Na^+^ + OH^−^ + H^+^ + OCl^−^

HOCl interacts with the major classes of biomolecules (i.e., amino acids, proteins, nucleotides, nucleic acids, carbohydrates, and lipids) and inorganic compounds to form free radicals [2]. HOCl exposure commonly damages proteins, DNA, and lipids [2]. Proteins are also reversibly modified by S-thiolation in the presence of HOCl [9]. Particularly destructive is the oxidation of ferrous ion (Fe^2+^) by HOCl to form hydroxyl radical (•OH), chloride ion (Cl^−^), and ferric ion (Fe^3+^) [2], with the latter being a catalyst of damaging Fenton chemistry [10].

Fe^2+^ + HOCl ⇨ Fe ^3+^ + •OH + Cl^−^

Genome-wide en masse insertion mutagenesis is an efficient means to discover gene functions. Recently, the approach was developed for use in *Haloferax volcanii* [11], a model archaeon isolated from the Dead Sea [12]. The strategy is broadly applicable and employs efficient in vitro transposition reaction of phage Mu [13] in combination with random in vivo gene targeting via homologous recombination to generate a mutant library [14]. 

Our prior work demonstrated that *H. volcanii* responds to hypochlorite stress in a manner that can be quantified at the proteome level by stable isotope labeling in cell culture (SILAC) coupled with tandem mass spectrometry analysis (LC-MS/MS) [15]. To further understand these responses on a global scale, we now report the development of an approach to select for *H. volcanii* mutants that are tolerant of extreme doses of NaOCl on a defined medium. The mutants were selected from a previously described comprehensive random transposon insertion library of *H. volcanii* [11]. The strains were selected for growth on high doses of NaOCl when using glycerol as the carbon/energy source, an organic alcohol common to hypersaline ecosystems [16]. The locations of the transposons on the genome were identified by inverse-nested two-step PCR (INT-PCR) and semi-random two-step PCR (ST-PCR). A selection of markerless deletion (transposon minus) strains, each with a disrupted gene identified in our analysis, was used to further define the cellular mechanisms of hypochlorite tolerance. An isogenic Δ*psmA1* (α1) mutant that produced only the α2 and β proteins of 20S proteasomes was found to be hypertolerant to hypochlorite. Thus, the type of α protein that forms the gate and outermost ring of 20S proteasomes can alter stress responses in this archaeon. 

## 2. Materials and Methods 

### 2.1. Materials

Biochemicals were from Sigma Aldrich (St. Louis, MO, USA). Other inorganic and organic analytical grade chemicals were from Fisher-Scientific (Atlanta, GA, USA). Klenow and other DNA polymerases, restriction endonucleases, and T4 DNA ligase were from New England Biolabs (Ipswich, MA, USA). Agarose for DNA analysis was from Bio-Rad laboratories (Hercules, CA, USA). Desalted oligonucleotide primers were purchased from Integrated DNA Technologies (Coralville, IA, USA). Reagent grade NaOCl solution (available chlorine 10%–15%, 425044–250 mL) was purchased from Sigma Aldrich.

### 2.2. Strains and Media 

Strains and primers used in this study are listed in Table S1. *H. volcanii* strains were grown at 42 °C at 200 rpm orbital shaking in glycerol minimal medium (GMM) with ammonium chloride used as the nitrogen source, as previously described [17]. Uracil was added at a concentration of 50 μg/mL for all Δ*pyrE2* strains. Growth in liquid medium was measured by optical density at 600 nm (OD_600_). The solid GMM (+uracil) medium was supplemented with 20 g/L agar (Sigma-Aldrich, catalog number: A7002). *H. volcanii* cells were incubated on agar plates in closed zippered bags at 42 °C for 5–10 days in the dark. American Type Culture Collection (ATCC) medium 974 [17] was only used for experiments that compared H26 and markerless deletion strains (not the transposon mutants) by liquid assay (see later section).

### 2.3. Isolation of Mutants with Enhanced Tolerance to Hypochlorite Stress 

To isolate strains with enhanced tolerance to hypochlorite stress, a transposon mutant library of *H. volcanii* H295 [11] was plated on increasing doses of NaOCl using GMM supplemented with uracil (+uracil) to compensate for the Δ*pyrE2* mutation of the parent strain (H295). The medium was devoid of tryptophan to stably maintain the transposon, which carried the tryptophan synthase gene (*trpA*). *H. volcanii* H26 (a *trpA*+ derivative of H295) was included as a control. The *H. volcanii* H295 transposon library was multiplied as previously described [18], stored in 20% glycerol at −80 °C, and thawed upon use. The cell mixture (10 μL) was diluted with 990 μL GMM (+uracil). Aliquots (100 μL) of the diluted cells were spread onto GMM (+uracil) agar plates supplemented with 0 to 1.2 mM NaOCl. Colony forming units (CFUs) per ml of aliquot were determined based on growth at 0 mM NaOCl. The plates were incubated at 42 °C for five days. Transposon mutant strains that grew at 1.2 mM NaOCl were streaked for isolation on GMM (+uracil) and stored at −80 °C in 20% (v/v) glycerol stocks. 

### 2.4. Genomic DNA Isolation 

Mutants that grew on GMM (+uracil) agar plates in the presence of 1.2 mM NaOCl were transferred into 3 mL GMM (+uracil) (in 13 × 100 mm tubes) and grown to mid-log phase (OD_600_ 0.6–0.8) at 42 °C with rotary shaking (200 rpm). Cells were pelleted at 14,000× *g* for 10 min (25 °C). The culture broth was removed, and the cell pellets were stored at −80 °C. Genomic DNA was extracted from the pellets by spooling [19]. 

### 2.5. Identification of Transposon Insertion Sites 

**Figure 1:**
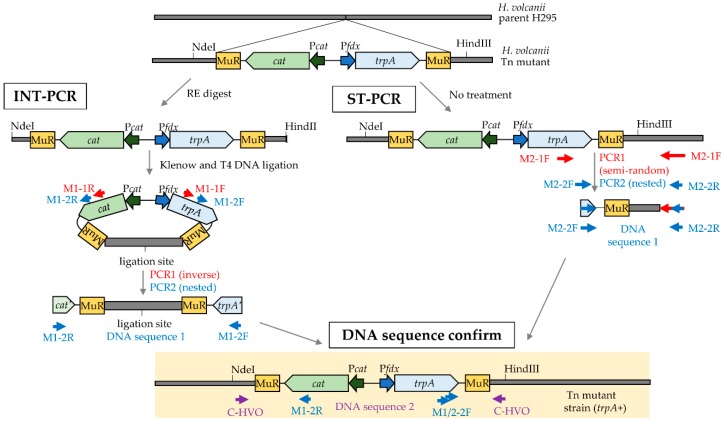
Schematic diagram of the inverted-nested two-step PCR (INT-PCR, left) and the semi-random two-step PCR (ST-PCR, right) strategies to identify the transposon insertion sites in *Haloferax volcanii*. The transposable (Tn) element includes the following: two Mu repeats (MuR), a chloramphenicol acetyltransferase (*cat*) gene, a P*cat* promoter, a tryptophan synthase (*trpA*) gene, and a ferredoxin promoter (P*fdx*). NdeI and HindIII are examples of the restriction enzyme (RE) sites used to cleave the genomic DNA prior to blunt-end ligation to form the circular DNA template used in the INT-PCR method. Primer pairs used for the two PCR steps (PCR1 and PCR2) and the DNA sequencing are color coded (red, blue, and purple) and numbered according to Table S1. See Methods for details.

Two PCR-based methods were used to identify transposon insertion sites on the *H. volcanii* genome as outlined in Figure 1. 

#### 2.5.1. Inversed Nested Two-Step PCR 

Genomic DNA was digested with restriction enzymes, including the following: (i) NdeI and HindIII, (ii) BmtI and BspHI, (iii) NdeI and NheI, or (iv) XhoI and BclI. The restriction enzymes were randomly selected and paired based on optimal activity in a common reaction buffer and temperature, but all were unable to cleave the transposon. The resulting genomic DNA fragments were treated with Klenow DNA polymerase to allow for the fill-in of 5’ overhangs and removal of 3’ overhangs. The Klenow-treated DNA was circularized by blunt-end ligation using T4 DNA ligase. The circularized DNA was used as a template for the INT-PCR approach. In the first (inverse PCR) step, primer M1-1F and M1-1R were designed to anneal to the *trpA* and *cat* genes of the transposon, respectively, and generate a DNA product that carried a portion of the transposon along with the genomic DNA that was adjacent to transposon insertion site. In the second (nested PCR) step, the M1-2F/2R primers were designed to target and amplify the inverse PCR product. 

#### 2.5.2. Semi-Random Two-Step PCR 

ST-PCR was performed according to the work of [20] with the following modifications. In the first PCR step, primer M2-F1 was designed to specifically anneal to the *trpA* gene of the transposon, while the 3’ end of the degenerate, primer M2_R1 was used to randomly anneal to the *H. volcanii* genomic DNA including the region adjacent to the transposon insertion site. The DNA product generated by the first PCR was subsequently used as a template for nested PCR, with the M2-2F primer being specific to the 3’ end of the *trpA* gene of the transposon and M2-2R primer being specific to the 5’ end of primer 2.

#### 2.5.3. DNA Sequencing to Identify the Transposon Insertion Sites 

The forward and reverse strands of the final DNA products generated by INT-PCR and ST-PCR were sequenced using the nested PCR primers (M1- and M2-2F/R, respectively) by the Sanger method (Eton Bioscience, Inc. San Diego, CA, USA). These DNA sequences (DNA sequence 1, Figure 1) were compared to the *H. volcanii* DS2 genome by NCBI BLAST nucleotide and blastx [21] to determine the transposon insertion site. A confirmation primer (C_HVO locus tag number) that specifically annealed to the genomic region adjacent to the transposon insertion site was also paired with an appropriate nested PCR primer (M1-2F/R or M2-F) to generate a ‘confirmation PCR product’ using genomic DNA isolated from the mutant strain as a template. The PCR product was excised from the gel and purified with QIAquick Gel Extraction Kit (Qiagen, Germantown, MD, USA), following which each strand of the DNA was analyzed by Sanger sequencing using the primer pairs of the confirmation PCR (DNA sequence 2, Figure 1).

### 2.6. PCR Conditions 

Phusion and OneTaq DNA polymerases were used for INT-PCR and ST-PCR, respectively (New England Biolabs). PCR (50 μL) reactions were mixed on ice with dimethyl sulfoxide (DMSO), buffer, deoxynucleotide triphosphate mix, primers (Table S1), template (spooled genomic DNA or PCR product), and DNA polymerase according to the supplier (New England Biolabs). PCR was performed using Mycyler and Icycler thermal cyclers (Bio-Rad) at the temperatures and times of incubation indicated in Table S2.

### 2.7. RNA Isolation and Real-time Quantitative Reverse Transcription PCR

Total RNA was isolated from *H. volcanii* cells by using the RNeasy minikit (Qiagen) according to the supplier’s instructions. DNA was removed by using a Turbo DNA-free kit (AM1907, Thermo Fisher Scientific, Waltham, MA USA) according to the recommendations of the supplier. The level of contaminating DNA after Turbo DNase digestion was below the limit of detection by PCR. The integrity of the RNA was determined by 2.0% (wt/vol) agarose gel electrophoresis. RNA (50 ng) per reaction mixture volume (50 μL) served as the template. One-step real-time quantitative reverse transcription PCR (qRT-PCR) was performed using the QuantiTect SYBR green RT-PCR kit (Qiagen) following the protocol described in the handbook of the supplier. The qRT-PCR procedure was performed under conditions of 50 °C for 30 min; 95 °C for 15 min; and 40 cycles of 95 °C for 15 s, 51 °C for 30 s, and 72 °C for 30 s, followed by determination of the melting curve using a CFX96 real-time C1000 thermal cycler (Bio-Rad). A single peak revealed by melting curve analysis indicated a single product. The messenger RNA (mRNA) levels were normalized to the internal standard *ribL* (*hvo_1015*). A standard curve was generated by using a QuantiTect SYBR green PCR kit (Qiagen) following the manufacturer’s protocol. Purified H26 genomic DNA served as the templates to test different primer pairs for PCR efficiency. Primers with PCR efficiencies between 95% (HVO_2469) and 101% (HVO_1957) were used (Table S1).

### 2.8. Hypochlorite Stress Plate Assay 

*H. volcanii* strains were streaked from −80 °C glycerol stocks onto GMM (+uracil) agar. The cells were incubated at 42 °C for five days. Isolated colonies were patched on GMM (+uracil) agar supplemented with 0, 0.8, 1.2, or 1.6 mM NaOCl as indicated. Cells were monitored for growth at 42 °C. 

### 2.9. Hypochlorite Stress Liquid Assay

*H. volcanii* markerless deletion and wild type strains were streaked onto ATCC 974 agar from −80 °C glycerol stocks. The cells were grown for five days at 42 °C. Isolated colonies were inoculated into 25 mL of ATCC 974 medium in 125 mL Erlenmeyer flasks and incubated at 42 °C with rotary shaking (200 rpm). At log phase (OD_600_ of 0.6–0.8), cells were washed twice with GMM (+uracil) by centrifugation (8600× *g*, 1 min at room temperature) and diluted to an OD_600_ of 0.1 unit in GMM (+uracil) supplemented with 0 or 1.5 mM NaOCl as indicated. The sample (150 μL) was transferred into a 96-well plate. The plate was covered with a lid and sealed with micro-pore tape to protect cells from desiccation. The cells were incubated at 42 °C (807 cycles-per-minute (cpm) shaking) in a micro plate reader (Epoch 2, BioTek, Winooski, VT, USA) with monitoring every 2 h at OD_600_. 

### 2.10. SDS-PAGE and Immunoblotting Analysis 

*H. volcanii* strains were streaked with a sterile toothpick from −80 °C glycerol stocks onto GMM (+uracil) agar and incubated at 42 °C for five days. Isolated colonies were transferred into 4 mL GMM (+uracil) (in 13 × 100 mm culture tubes) and grown with orbital shaking (at 200 rpm) for two days at 42 °C to an OD_600_ of 0.6. Cells were subcultured and similarly grown to an OD_600_ of 1.2. Cultures (1 mL) were harvested by centrifugation (16,873× *g* for 10 min at room temperature). The cell pellets were resuspended to a final OD_600_ of 0.065 per 10 μL by addition of 150–200 μL of 2× reducing SDS loading buffer (50 mM Tris-Cl buffer at pH 6.8 with 2% (w/v) SDS, 10% (v/v) glycerol, 0.3 mg·mL^−1^ bromophenol blue, and 2.5% (v/v) β-mercaptoethanol). Samples were boiled for 10 min. Proteins (10 uL sample) were separated by reducing 10% SDS-PAGE (sodium dodecyl sulfate polyacrylamide gel electrophoresis). Equivalent protein loading was based on OD_600_ of the cell culture (0.065 units per lane) and confirmed by Coomassie blue R-250 staining of parallel gels. Unstained proteins were electroblotted from the gels onto PVDF (polyvinylidene fluoride) membranes (Amersham) as per standard protocol (BioRad). Proteasome α1 subunit was detected using a 1:5000 dilution of an anti-α1 rabbit polyclonal antibody [22] followed by goat anti-rabbit IgG-HRP (horseradish peroxidase) Cruz Marker compatible antibody (SC-2030, Santa Cruz Biotechnology, Dallas, TX, USA) at a 1:1000 dilution. Immunoreactive antigens were detected using the Pierce enhanced chemiluminescence (ECL) Plus Western blotting substrate (Thermo Fisher Scientific) and Amersham Hyperfilm ECL (GE Healthcare Bio-Sciences, Pittsburgh, PA, USA). 

### 2.11. Prediction of Protein Structure and Function 

To discern biological mechanisms that may be used by haloarchaea to withstand hypochlorite stress, the function of the genes disrupted in the NaOCl-hypertolerant mutant strains was predicted as follows. Genes encoding proteins that had orthologs with a known function were identified by BlastP [21] and Interpro [23]. Signal peptide (Sec, Tat and lipobox) motifs and transmembrane spanning helices were predicted by SignalP 4.1 [24], TatP 1.0 [25], TatFind 1.4 [26], PSORTb 3.0 [27], and TMHMM 2.0 [28]. A 3D protein structure was modeled using Phyre 2.0 [29]. Conserved active sites/motifs were identified by comparison of the 3D protein models to biochemically characterized proteins using Chimera 1.11 [30]. Operon organization and genome synteny were analyzed using the UCSC (University of California Santa Cruz) Archaeal Genome Browser [31] and SyntTax [32]. 

## 3. Results and Discussion

### 3.1. *Haloferax volcanii* Mutants of Enhanced Tolerance to Hypochlorite Stress 

**Figure 2:**
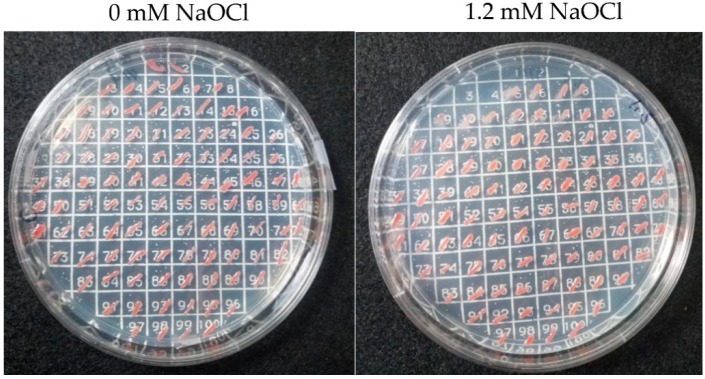
*H. volcanii* transposon mutant strains were found to be hypertolerant to hypochlorite stress. NaOCl-hypertolerant strains (positions 5–100) were compared to H26 (DS70 Δ*pyrE2*, position 1), HM1041 (H26 Δ*samp2*, position 2), a transposon library pool (position 3), and HM1052 (H26 Δ*ubaA*, position 4). Cells were patched on glycerol minimal medium (GMM) (+uracil) with 0 mM NaOCl (left) and 1.2 mM NaOCl (right) for the comparison. See Methods for details.

To identify *H. volcanii* mutants of enhanced tolerance to hypochlorite stress, a transposon mutant library was compared to wild type (H26) for growth in the presence of increasing doses of NaOCl. GMM (+uracil) agar plates supplemented with 1.2 mM NaOCl were found to clearly distinguish wild type (H26) from mutant strains when examining 3–5 × 10^6^ CFUs per plate. No growth was observed for the H26 control under these conditions. By contrast, the transposon mutant library yielded ~100 CFUs per plate, thus reaching a survival rate of 0.0025%. Individual colonies of the transposon mutant library were further isolated on the selective medium and demonstrated to be tolerant of at least 1.2 mM NaOCl, when compared twith H26 and other strains, which did not survive under these conditions (Figure 2). 

### 3.2. Transposon Insertion Sites Mapped on the Genome of *Haloferax volcanii* Mutant Strains were Found to be Hypertolerant to Hypochlorite 

**Figure 3:**
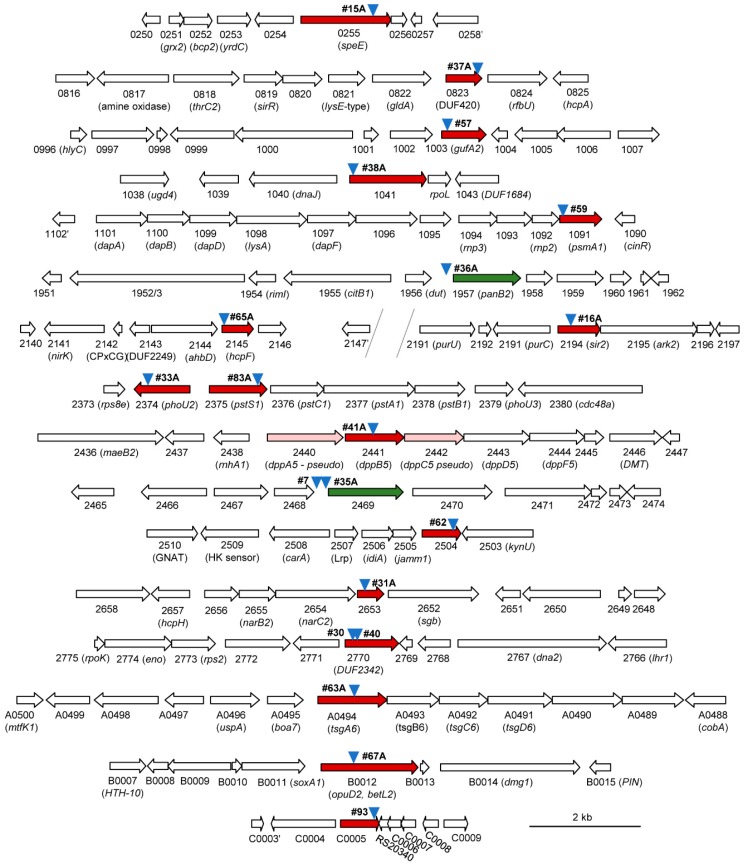
Schematic diagram of the genomic neighborhood of the transposon insertion site of the *H. volcanii* mutants that were hypertolerant to hypochlorite. Down arrowheads (blue) represent the site of transposon insertion with the strain isolate number indicated adjacent to the symbol. Arrows represent open reading frames (ORFs) deduced from the *H. volcanii* genome sequence (UniProt proteome ID: UP000008243). HVO_ gene locus tag number and select gene names are indicated below the ORF. Genes with transposons located within the ORF are in red. Pseudo genes are in pink. Genes in green are downstream of transposons that are inserted within intergenic regions. Scale bar, 2 kb.

**Figure 4:**
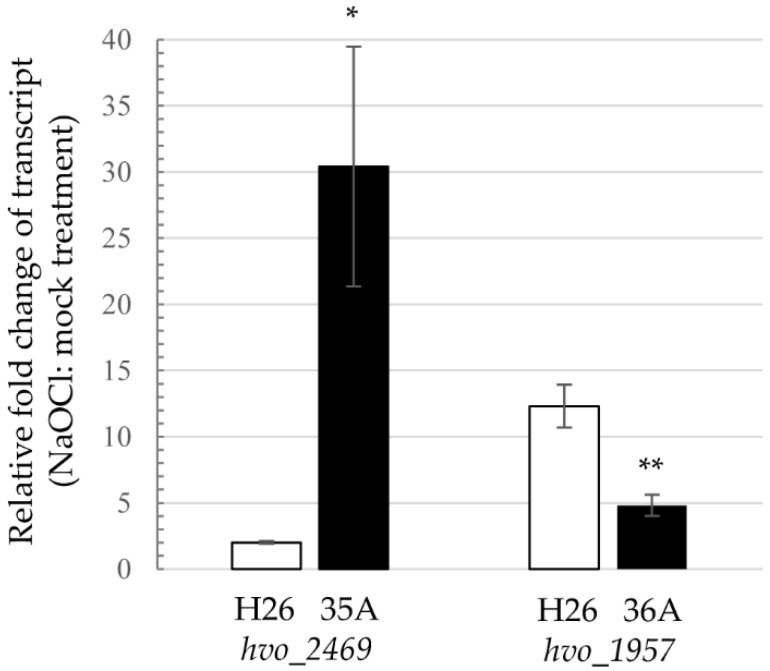
Transcript level responses to NaOCl stress are altered by transposon insertions 5’ of the TATA box of *hvo_2469* and *hvo_1957*. *H. volcanii* strains were grown to exponential phase and treated with 0 and 3 mM NaOCl for 60 min. Total RNA was extracted and used for real-time quantitative reverse transcription PCR (qRT-PCR). Gene expression levels were normalized to the internal reference *ribL* (one-fold). *H. volcanii* strains and the qRT-PCR gene targets are indicated below the *x*-axis. Significant differences between the wild type (H26) and mutant (35A and 36A) strains were calculated by Student’s *t*-test analysis with equal variance (*p*-value < 0.05 * and *p* < 0.01 **). All data are expressed as mean ± standard error of the mean (SEM) for *n* = 3 technical replicates. See Methods for details.

Two basic approaches (INT-PCR and ST-PCR) were used to map the transposon insertion sites (Figure 2). These approaches, which differed from the whole genome sequencing method previously reported to map the transposon insertion sites [11,18], were found to be useful in the rapid identification of the transposon insertion site of twenty distinct isolates (Figure 3, Table S3). Fourteen of the sites were identified by INT-PCR, while six sites were identified by ST-PCR. The ST-PCR method was found to be more rapid but less prone to positive identification. Most of the mutant strains (17 of 20 total) had transposons inserted within an open reading frame (ORF), suggesting the loss of gene function. Eleven of these sites were linked to genes that, in wild type (H26) cells, encode proteins detected by SILAC-based LC-MS/MS analysis [15], including three proteins (HVO_2375, HVO_1041, and HVO_1957) of significant differential abundance, after NaOCl stress (see later discussion for details). Three of the isolates had a transposon inserted within an intergenic region upstream (5’) of the predicted TATA box promoter element (isolates 7, 35A, and 36A; Figure S1). This intergenic positioning of the transposon (which has internal promoter elements, P*cat* and P*fdx*) was speculated to have altered the expression of the downstream genes (*hvo_2469* in isolates 7 and 35A and *hvo_1957* in isolate 36A). Thus, the expression of these genes was monitored by qRT-PCR and was found to be significantly altered in response to NaOCl in the mutants compared with wild type (H26) (Figure 4 and later discussion). In addition to the intergenic insertions, several of the clones harbored transposons in the same genomic region as exemplified by two insertions 5’ of *hvo_2469* (isolates 7 and 35A), two insertions in the *hvo_2374–2375* region (isolates 33A and 83A), and two insertions in *hvo_2770* (isolates 30 and 40) (Figure 3).

### 3.3. Membrane versus Intracellular Functions 

More than half (11/20) of the hypertolerant mutant strains had transposon insertions within or adjacent to genes predicted to encode proteins associated with the membrane (Table S3). By comparison, only 24% of the theoretical proteome is estimated to be membrane proteins [33]. Several of the mutant strains were disrupted in ORFs predicted to encode pre-proteins (Figure S2) that would be translocated through the twin arginine translocation (Tat) system, cleaved by a protease to expose an N-terminal cysteine and lipid modified at this cysteine residue [34]. Thus, lipoprotein maturation may be generally sensitive to oxidative stress, as this process occurs within the cell membrane and requires a cysteine thiol group. Of the membrane-associated ORFs that were disrupted by the transposon insertions, most were involved in transport (HVO_1003, HVO_2374, HVO_2375, HVO_2441, HVO_2469, HVO_A0494, HVO_B0012), with others related to redox homeostasis (HVO_0823 and HVO_2145), spermidine synthase (HVO_0255), or unknown functions (HVO_2653) (see later discussion). 

### 3.4. Metal Ion Transport 

Gene homologs of metal ion transport were found to be disrupted in cells hypertolerant to hypochlorite stress. HVO_1003, a member of the zinc transport protein (ZIP) family that function in the uptake of zinc and/or other metals [35], was disrupted in isolate 57 (Figure 3). During oxidative stress, zinc can replace the Fe^2+^ released from damaged Fe–S clusters and inactivate these metalloenzymes, thus causing metabolic bottlenecks [36,37]. Reduced levels of intracellular zinc may be the mechanism that enables the *hvo_1003*::Tn mutant to be at a selective advantage over the wild type when challenged with NaOCl. HVO_2441, a homolog of the ATP-binding cassette (ABC) permease DppB that functions in the uptake of dipeptides and heme-iron in bacteria [38], was also found to be disrupted in a NaOCl hypertolerant mutant (Figure 3). Impaired heme-based iron transport is predicted to reduce the intracellular pool of labile iron that causes damaging Fenton chemistry during oxidative stress [39]. Interestingly, *hvo_2441* (*dppB5*) is flanked by two pseudo genes (*dppA5* and *dppC5*) (Figure 3), suggesting the function of this transport system is already reduced in *H. volcanii* DS2 derived strains such as H26 [40]. 

### 3.5. Inorganic Phosphate Transport 

Gene homologs associated with inorganic phosphate (Pi) regulation (HVO_2374, PhoU2) and transport (HVO_2375, PstS1) were disrupted in two of the NaOCl-hypertolerant strains (Figure 3). Consistent with this finding, PstS1 abundance is down during NaOCl stress in *H. volcanii* [15]. PstS1 is one of the two solute binding protein homologs in haloarchaea that are associated with the ABC-type Pi uptake system operons *pts1* (*pstS1C1A1B1*) and *pts2* (*pstS2C2A2B2*) [41]. Like PstS2, PtsS1 was found to have the conserved residues needed to coordinate and facilitate Pi uptake (Figure S3). In *Halobacterium salinarum*, Δ*pts1* mutants have a higher rate of Pi uptake than Δ*pst2* mutants [41], suggesting the NaOCl-hypertolerant *ptsS1*::Tn mutant had increased intracellular levels of Pi. The *H. volcanii* PhoU2 protein, by contrast, was predicted to be a transcriptional regulator of Pi uptake as it was found to have an N-terminal DNA binding domain and to be in synteny with the *pts1* operon; it also was found to harbor the conserved residues needed to coordinate a multinuclear iron cluster (Figures S4 and S5) that is used in Pi uptake by comparison with characterized PhoU proteins [42]. In bacteria, disruption of *phoU2* increases expression of Pi transport and elevates levels of inorganic polyphosphate (polyP), an intracellular polymer that promotes hypochlorite tolerance [43]. Thus, the mechanism(s) of NaOCl-hypertolerance of the *H. volcanii phoU2*::Tn and *pstS1*::Tn isolates may be related by enhanced levels of intracellular Pi and/or polyP. 

### 3.6. Organic Molecule Transport 

Organic molecule transport homologs (HVO_A0494 (TsgA6), HVO_B0012 (BetT), and HVO_2469 (SNF)) were found to be disrupted in several NaOCl-hypertolerant strains (Figure 3). This finding was insightful as the growth medium included only five organic molecule supplements: glycerol (20 mM), thiamine (0.8 μg/mL), biotin (0.1 μg/mL), uracil (50 μg/mL), and tris(hydroxymethyl)aminomethane (Tris, 30 mM). 

TsgA6, a predicted Tat lipoprotein of the ABC-type solute binding protein family 1 (IPR006059), was found to be disrupted in isolate 63A (Figure S2). This family includes solute binding proteins that facilitate the uptake of maltose/maltodextrin (MalE/X), oligosaccharide (MsmE), glycerol-3-phosphate (UgpB), and thiamine (TbpA) [44]. While TsgA6 was related to MalE in the 3D structure, it was not predicted to bind maltose (Figure S6). A polar effect on transcription/translation cannot be ruled out, as *tsgA6* was the first gene of the *tsgA6B6C6D6* ABC-transport system operon (Figure 3). One explanation is that the transposon insertion in *tsgA6* rendered cells hypertolerant to hypochlorite by minimizing the synthesis of unnecessary organic molecule transporters that span the membrane, as glycerol was the sole carbon/energy source of the selective conditions.

HVO_B0012, a member of the betaine/carnitine/choline transporter (BCCT) family and the amino acid–polyamine–organocation superfamily (APCS), was found to be disrupted in the NaOCl-hypertolerant strain 67A (Figure 3). BCCT family members mediate the Na^+^/H^+^-coupled symport or precursor/product antiport of organic molecules with positively charged nitrogen or sulfur head-groups [45,46]. HVO_B0012 was found to be 43% identical to the H^+^-driven betaine/choline transporter BetT and in genome synteny with homologs of betaine/choline metabolism and oxidative stress response (Figure S7). While bacteria produce, excrete, and reaccumulate betaine/choline during osmotic stress [47], the salt-in strategy of *H. volcanii* and growth on GMM (+uracil) may have alleviated the need for HVO_B0012 during hypochlorite stress. Betaine/choline metabolism in *H. volcanii* was predicted to require electron transfer and H_2_O_2_ generation (which may exacerbate ROS damage) (Figure S7). 

The third organic molecule transporter identified in the transposon library screen was HVO_2469, a member of the sodium neurotransmitter symporter family (SNF, IPR000175), and close homolog of MhsT, a Na^+^-dependent transporter of hydrophobic L-amino acids [48] (Figure S8). Unlike the other transporters that were disrupted in the coding sequence, the site of transposon insertion was 5’ of the BRE (B recognition element) and TATA box promoter elements of *hvo_2469* (Figure S1). Further analysis by qRT-PCR revealed that *hvo_2469* transcripts were up 2-fold in the wild type and over 30-fold in isolate 35A during hypochlorite stress (Figure 4). This result suggested the SNF transporter homolog HVO_2469 was more abundant in the hypertolerant mutant compared with the wild type during hypochlorite stress. One possible explanation is that the transporter facilitated the re-accumulation/uptake of hydrophobic amino acids, known to have strong radical scavenging activities [49]. 

### 3.7. Polyamine Synthesis 

The polyamine aminopropyltransferase homolog HVO_0255 (SpeE) was found to be disrupted in the hypertolerant mutant 15A (Figure 3). Polyamines are polycationic molecules that interact with negatively charged regions of biomolecules (e.g., nucleic acids, lipids, and proteins) [50] and are considered ‘primordial stress molecules’ based on their ability to protect cells from oxidative damage or induce oxidative stress [51]. In thermophiles, differences in polyamine ratios are correlated with growth temperature [52]. *H. volcanii* produces the polyamines agmatine and cadaverine [53,54] and has two SpeE homologs (HVO_B0357 and HVO_0255) that are predicted to be integral membrane proteins with conserved active site residues and structural homology to polyamine aminopropyltransferases (Figure S9). Interestingly, the *H. volcanii* SpeEs were found to differ in primary sequence at key regions known to alter polyamine binding specificity in thermophilic polyamine aminopropyltransferases [55]; HVO_0255 had a GG(GA)G(F/Y) motif and long C-terminal extension, while HVO_B0357 had a GGGD(W/Y) motif and short-C-terminal tail (Figures S9 and S10). Thus, the haloarchaeal SpeEs are predicted to have distinct polyamine binding specificities, such that the *hvo_0255* mutation would alter the polyamines ratios that promote NaOCl-hypertolerance.

### 3.8. Membrane Associated Redox Reactions 

Several ORFs associated with redox reactions in the cell membrane were found to be disrupted in the hypertolerant strains, including *hvo_2145* (*hcpF*), *hvo_0823*, and *hvo_2653* (Figure 3). Halocyanins, such as HcpF, are blue (type-1) copper proteins that serve as mobile electron carriers in the peripheral membrane of haloarchaea [56,57]. HcpF is one of eight halocyanins (HcpA-H) predicted to be translocated by the Tat system and one of four (HcpC, HcpD, HcpE, and HcpF) that is a putative Tat lipoprotein (Figure S2). Disruption of *hcpF* may have shifted the cell to halocyanins that are more tolerant of oxidant and/or less prone to transfer electrons to complexes that form oxygen radicals, such as cytochrome oxidase [58,59]. The *hvo_0823*, disrupted in isolate 37A, encodes a cytochrome *c*-oxidase (EC: 1.9.3.1) type helical bundle protein homolog related in structure to the non-heam binding multipass transmembrane domain of *caa3*-type cytochrome oxidases (Figure S11). These results suggest that HVO_0823 could impact the integrity and/or maturation of the *H. volcanii* cytochrome oxidase(s) [60] and, thus, reduce ROS production. The ORF of the multipass transmembrane domain protein HVO_2653 was also found to be disrupted in one of the hypertolerant strains; *hvo_2653* is in genomic synteny with halocyanin (*hcpH*) and nitrate reductase (*narB2* and *narC2*) genes (Figure 3), suggesting it is associated with redox reactions in the cell membrane. Overall, the transposon insertions in *hcpF*, *hvo_0823*, and *hvo_2653* may have shifted electron transfer from enzymes that leak electrons and radicals to systems that avoid metabolic bottlenecks during hypochlorite stress.

### 3.9. Oxidoreductase and Hydrolase Enzymes 

Several soluble oxidoreductase and hydrolase homologs were found to be impaired in hypertolerant mutant strains. Isolate 62 was disrupted in *hvo_2504* encoding a member of the NAD(P)^+^-dependent short-chain dehydrogenase/reductase (SDR) family (IPR002347), which was found to be distinct from characterized SDRs based on the absence of conserved catalytic tetrad residues (Figure S12). Isolates 30 and 40 were disrupted in *hvo_2770*, encoding a member of the MA clan of zinicin-like metalloproteases (MEROPS peptidase database, http://www.ebi.ac.uk/merops/) that had the conserved residues to coordinate the catalytic Zn^2+^ ion (Figure S13). While zinicins function in biological processes, such as cell signaling [61], which may explain the enhanced tolerance of isolates 30 and 40 to hypochlorite, the transposon insertions were also positioned at 5’ of *hvo_2771* encoding a glyoxalase-like gene homolog. Enhanced levels of glyoxalase I are associated with tolerance to oxidative/hypochlorite stress [62,63], which could also be the rationale for our findings. The NaOCl-hypertolerant isolate 93 was found to be disrupted in *hvo_c0005*, encoding a member of the six-hairpin glycosidase-like (IPR008928) superfamily, including enzymes that synthesize/break glycosidic bonds damaged by hypochlorite [2]. Isolate 38A was disrupted in *hvo_1041*, encoding a metallo-β-lactamase homolog that had the conserved active site residues needed to coordinate the two catalytic Zn^2+^ ions (Figure S14). HVO_1041 was most closely related to *Thermus thermophilus* TTHA1429 with an unknown function [64], based on 3D structural homolog modeling (Figure S14), and was predicted to cleave RNA based on genomic synteny with *rpoL* (DNA-directed RNA polymerase subunit L) (Figure 3). However, our previous SILAC-based proteomics reveals HVO_1041 to be up in abundance after exposure to NaOCl [15], suggesting that disruption of this gene was not responsible for the observed NaOCl hypertolerance. Instead, we propose that the transposon insertion caused an upregulation of the adjacent gene *hvo_1040*, encoding a DnaJ (Hsp40) chaperone domain protein that facilitated protein folding.

### 3.10. Protein Lysine Deacetylation 

The NAD^+^-dependent histone deacetylase (HDAC) family protein Sir2 (HVO_2194) of the Gcn5-related N-acetyltransferase (GNAT) subfamily was found to be disrupted in the hypochlorite tolerant strain 16A (Figure 3). *H. volcanii* encodes two HDACs (the class III Sir2 and the class II HdaI) and three histone acetyltransferase (HAT) family homologs (Pat1, Pat2, and Elp3). Of these, HdaI is essential for growth [65], and Pat1/2 are inversely correlated in protein abundance during hypochlorite stress (Pat1 is down 3.1-fold, while Pat2 is up 1.8-fold) [15]. Thus, enzymes that control lysine acetylation are linked to hypochlorite stress. In *Sulfolobus* sp., Sir2 deacetylates the chromatin protein Alba, resulting in Alba binding to the chromosome and transcriptional repression [66]. While Alba-like superfamily (IPR036882) proteins are not conserved in *H. volcanii*, a wide variety of proteins are found to be lysine acetylated in *Haloferax mediterranei* [67]. The *sir2*::Tn mutation of isolate 16A is presumed to stabilize proteins in their acetylated state. The addition of an acetyl group could generally block and protect the amino groups of lysine residues, which are particularly susceptible to free radical formation by HOCl [2]. Alternatively, lysine acetylation may be associated with specific pathway(s), such as chromatin remodeling, DNA replication repair, and/or metabolism, to overcome hypochlorite stress.

### 3.11. Proteasome Components 

**Figure 5:**
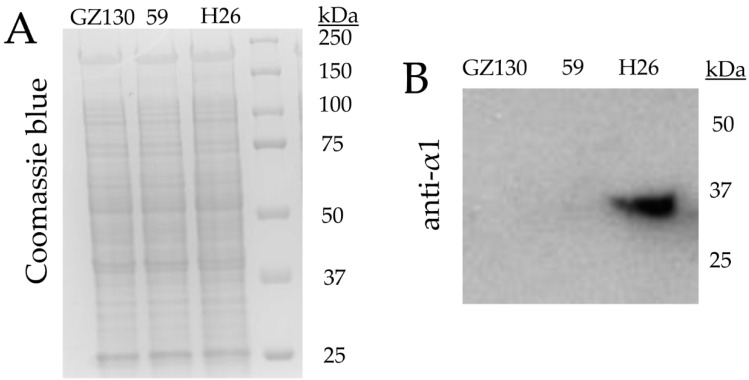
The 20S proteasomal α1 protein is not detected in one of the strains (isolate 59) that was hypertolerant to hypochlorite stress. Whole cells lysate, separated by reducing 10% SDS-PAGE (sodium dodecyl sulfate polyacrylamide gel electrophoresis), was analyzed for total protein by Coomassie Blue staining (**A**) and α1 protein by immunoblotting (**B**). Strains used for analysis included H26 (parent strain), GZ130 (Δ*psmA1*), and isolate 59 (*psmA1*::Tn) as indicated. See Methods for details.

**Figure 6:**
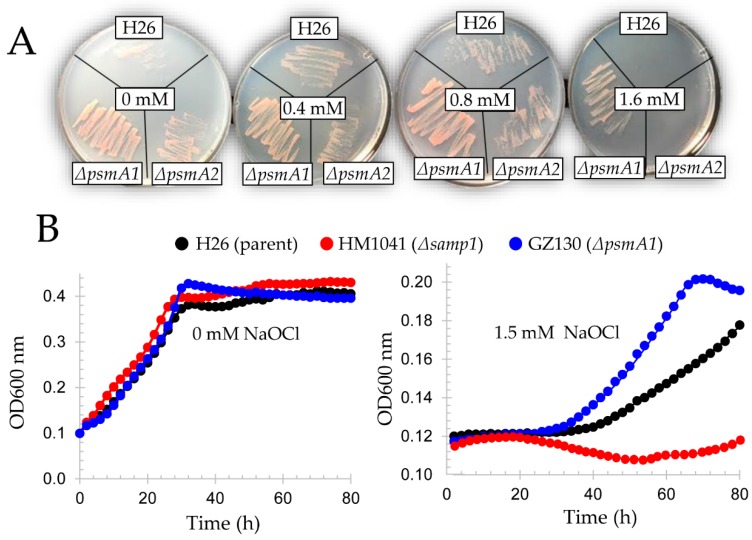
Markerless deletion of *psmA1* renders cells hypertolerant to hypochlorite stress. *H. volcanii* strains H26 (parent), GZ130 (Δ*psmA1*), GZ114 (Δ*psmA2*), and HM1041 (Δ*samp1*) were analyzed for tolerance to hypochlorite by plate (**A**) and/or liquid (**B**) assay. *H. volcanii* cells are more tolerant of NaOCl in liquid culture compared with agar plates. Experiments were performed in experimental and technical triplicate and are presented as an average. See Methods for details.

Isolate 59 was found to have a transposon insertion in *psmA1* (*hvo_1091*) encoding proteasomal subunit α1, while isolate 36A had a transposon inserted at 5’ of *panB1* (*hvo_1957*) encoding a proteasomal Rpt-like AAA ATPase (Figure 3). Archaea encode different types of 20S proteasomes and AAA ATPases that form a proteasome system network [68,69]. In the case of *H. volcanii*, the α1 and α2 proteins associate with β in combinations of α1β, α2β, and α1α2β to form at least three distinct types of 20S proteasomes [22,70,71]. While 20S proteasomes are essential for growth, the individual type of α subunit is not; in other words, *H. volcanii* requires the β subunit and at least one type of α subunit (α1 or α2) to grow [72]. By contrast, the AAA ATPase complexes of PanA1, PanA1/B2, and PanB2 are not essential for growth of *H. volcanii* [71,72]. Consistent with disruption of the *psmA1* gene, the α1 protein was not detected in the *psmA1*::Tn mutant (isolate 59) by immunoblotting analysis (Figure 5). Based on this finding, the isogenic Δ*psmA1* (*psmA*, *α1*) strain GZ130 was analyzed for hypertolerance to hypochlorite and was found to grow on GMM (+uracil) agar plates supplemented with 1.6 mM NaOCl; by contrast, the Δ*psmA2* (*psmC*, *α2*) (GZ114) and parent (H26) strains were unable to grow under these conditions (Figure 6A). In liquid culture, where higher concentrations of NaOCl are required for toxicity, the Δ*psmA1* mutant was found to be more tolerant of NaOCl than the parent (H26) and Δ*samp1* ubiquitin-like mutant (HM1041), with the latter being found to be hypersensitive to NaOCl (Figure 6B), as previously described [15]. The AAA ATPase PanB2 was also associated with NaOCl hypertolerance. After exposure to NaOCl, the *panB2* transcript levels were found to be up by more than 10-fold in the wild type (H26) and up 5-fold in isolate 36A, suggesting the transcript level stress response at this locus was dysregulated in the mutant (Figure 4). This apparent dysregulation may explain the hypertolerance of isolate 36A to hypochlorite stress. A previous study revealed that the levels of α2 and PanB2 were elevated during the stationary phase; whereas α1 and PanA1 were prevalent during log phase growth [71]. In addition, PanB2 was found to be up after exposure to NaOCl [15]. These changes in the composition of proteasome system apparently prepare the cell for stresses, such as hypochlorite and stationary phase, that are encountered in hypersaline environments. 

## 4. Conclusions

Here, we developed an assay to select and isolate *H. volcanii* mutant strains from a random transposon library that displayed enhanced tolerance to hypochlorite stress when grown on glycerol. We demonstrate that two economical and rapid PCR-based methods (INT-PCR and ST-PCR) can be used to identify the transposon insertion sites on the *H. volcanii* genome. Thus, whole genome sequencing is not required to identify the insertion sites. In the hypochlorite tolerant strains, transposon insertions were found within or upstream of genes associated with lysine deacetylation, proteasome components, transporters, polyamine biosynthesis, electron transfer, and other functions. qRT-PCR analysis revealed that transposons inserted at 5’ of promoter consensus sequences can perturb the transcript abundance of the downstream gene. Subsequent analysis of markerless deletion strains demonstrated that cells with 20S proteasomes composed of α2β are more tolerant of hypochlorite stress than those of α1β subunit composition or a combination thereof. Overall, this approach provided a global view of hypochlorite tolerance in the model archaeon *H. volcanii*. 

## Supplementary Materials

The following are available online at http://www.mdpi.com/2073-4425/9/11/562/s1, Figure S1: Transposon insertion sites with respect to the promoter region of *hvo_2469* (SNF family transporter) and *hvo_1957* (AAA-type ATPase proteasome associated nucleotidase PanB2); Figure S2: Twin arginine translocation (Tat) signal peptide and lipoprotein motifs of select *H. volcanii* proteins; Figure S3: *H. volcanii* PstS1 (HVO_2375) is structurally related to bacterial phosphate (Pi) solute binding proteins; Figure S4: PhoU domain family proteins of *H. volcanii*; Figure S5: Relationship of *H. volcanii* PhoU2 (HVO_2374) to *Thermotoga maritima* Tm1734; Figure S6: Comparison of *H. volcanii* TsgA6 (HVO_A0494) with the *E. coli* maltose binding protein (MalE); Figure S7: *H. volcanii* HVO_B0012 (BetT) and its predicted function in choline transport; Figure S8: *H. volcanii* SNF family transporter HVO_2469; Figure S9: *H. volcanii* SpeE homologs are related to the polyamine aminopropyltransfease of *Thermus thermophiles*; Figure S10: Multiple amino acid sequence alignment of the active site region of haloarchaeal members of the SpeE family; Figure S11: *H. volcanii* HVO_0823 is a cytochrome *c*-oxidase (EC: 1.9.3.1) type helical bundle protein; Figure S12: HVO_2504 is a putative oxidoreductase of the short-chain dehydrogenase/reductase (SDR, IPR002347) family; Figure S13: *H. volcanii* HVO_2770 is a DUF2342 protein of the zinicin-like metallopeptidase type 2 (IPR018766) and F420 biosynthesis associated (IPR022454) families; Figure S14: *H. volcanii* HVO_1041 is a metallo-β-lactamase superfamily protein; Table S1: Strains, plasmids, and oligonucleotide primers used in this study; Table S2: Cycling conditions for the inverse-nested two-step PCR (INT-PCR) and semi-random two-step PCR (ST-PCR); Table S3: Identification of transposon insertion sites on the genome of *H. volcanii* mutant strains are found to be hypertolerant of hypochlorite stress. Ref: [73,74,75,76,77,78,79].
