# Supplementary material for: Molecular Factors of Hypochlorite Tolerance in the Hypersaline Archaeon Haloferax volcanii
Source: Genes (Basel). 2018 Nov 20;9(11):562. doi: 10.3390/genes9110562 (PMC6267482; doi:10.3390/genes9110562)
Supplement: Supplementary file 1 [file genes-09-00562-s001.pdf]

Supplemental Data

Title: Molecular factors of hypochlorite tolerance in the hypersaline archaeon *Haloferax volcanii*

Authors: Miguel Gomez[1+], Whinkie Leung[1+], Swathi Dantuluri[1], Alexander Pillai[1], Zyan Gani[1], Sungmin Hwang[1], Lana J. McMillan[1,2], Saija Kiljunen[3], Harri Savilahti[4] and Julie A. Maupin-Furlow[1,2*]

Affiliations: [1]Department of Microbiology and Cell Science, Institute of Food and Agricultural Sciences, University of Florida, Gainesville, FL 32611, USA; [2]Genetics Institute, University of Florida, Gainesville, FL 32611, USA; [3]Department of Bacteriology and Immunology, Immunobiology Research Program, University of Helsinki, Helsinki, Finland; [4]Division of Genetics and Physiology, Department of Biology, University of Turku, Turku, Finland.

***Corresponding Author:** Department of Microbiology and Cell Science, University of Florida, Gainesville, FL 32611, USA. Phone: (352) 392-4095; Facsimile (352) 395-5922; Email: jmaupin@ufl.edu

[+]**Primary authors:** contributing equally to this work.

Contents

Supplemental Table S1. Strains, plasmids and oligonucleotide primers used in this study.

Supplemental Table S2. Cycling conditions for the inverse-nested two-step PCR (INT-PCR) and semi-random two-step PCR (ST-PCR).

Supplemental Figure S1. Transposon insertion sites with respect to the promoter region of *hvo_2469* (SNF family transporter) and *hvo_1957* (AAA-type ATPase proteasome associated nucleotidase PanB2).

Supplemental Figure S2. Twin arginine translocation **(**Tat) signal peptide and lipoprotein motifs of select *H. volcanii* proteins.

Supplemental Figure S3. *H. volcanii* PstS1 (HVO_2375) is structurally related to bacterial phosphate (Pi) solute binding proteins.

Supplemental Figure S4. PhoU domain family proteins of *H. volcanii*.

Supplemental Figure S5. Relationship of *H. volcanii* PhoU2 (HVO_2374) to *Thermotoga maritima* Tm1734.

Supplemental Figure S6. Comparison of *H. volcanii* TsgA6 (HVO_A0494) to the *E. coli* maltose binding protein (MalE).

Supplemental Figure S7. *H. volcanii* HVO_B0012 (BetT) and its predicted function in choline transport.

Supplemental Figure S8. *H. volcanii* SNF family transporter HVO_2469.

Supplemental Figure S9. *H. volcanii* SpeE homologs are related to the polyamine aminopropyltransfease of *Thermus thermophilus*.

Supplemental Figure S10. Multiple amino acid sequence alignment of the active site region of haloarchaeal members of the SpeE family.

Supplemental Figure S11. *H. volcanii* HVO_0823 is a cytochrome *c*-oxidase (EC: 1.9.3.1) type helical bundle protein.

Supplemental Figure. S12. HVO_2504 is a putative oxidoreductase of the short-chain dehydrogenase/reductase (SDR, IPR002347) family.

Supplemental Figure S13. *H. volcanii* HVO_2770 is a DUF2342 protein of the zinicin-like metallopeptidase type 2 (IPR018766) and F420 biosynthesis associated (IPR022454) families.

Supplemental Figure S14. *H. volcanii* HVO_1041 is a metallo-β-lactamase superfamily protein.

A)

```
HVO_2468 TGACC CGACTCCACC
         ▼CTCTCTGATT CGTGGCGACA TTCGCCACGA TAAGGGATGA CATTCGTTTC
          GGTGCGCACG AATTTTTACA CGGTACGCAA GTGATTCACG AACACGACAC
          TCGTTAGAAA AATGAGGCTA TTCGGGGGGC GAATTCGGAC GAACGAACCA
          TATATGATAG TTGGTTAGAG AATTGTGTCG AATTTTGCTC CGATATGCGA
         CGGCATAC▼CG CGTCATTTAT CACACCCACA TCGGCAACAG GGTGATAGAC ATG HVO_2469
                  CR nAAT TTTAW A
```

B)

```
HVO_1956 TGACG ACCGCCGACC GCGACTCTCG GAAGCGGTCA CTCGCCGGAT ACTACCGGAT
               GAATACCGAC ATCCCGATTT ACGGGACGTT CTCGGCGATG ACGAATTCCT
               TTCAAGTCGA TGGGCGTCGT ATCCCTTCTC GCGGAGGGCA TGTCGGGCCG
               AAACGAGGGG CGACCTGTAC CCACCGAACC TCCGCAACTT CCTCCTTCCA
               CAGCTTCGTC TTCGTTGCTT CGCGCGTTCC TGCGAACCGC TCGTGCCCGT
               GAGCCGCGGC GTCGCGCTGC CGGCCTGCGA TTCGCCGCCG CGAGCGAGCG
               CCACGGGACC GCCGCCACCT AAAGCCGCTT CGAGCGCGGC AGTCGGGTTC
              TCTGACCCAA AGG▼CACATTT ACGCGCATAC CTAAATGAGG CCAATCACTG ATG HVO_1957
                   CRnAA T        TTT AWA
                           CR nAAT
```

Figure S1. Transposon insertion sites with respect to the promoter region of *hvo_2469* and *hvo_1957*. Indicated are transposon insertion sites (▼), start/stop codons (red/green), and conserved promoter elements including the TATA box (TTTAWA, blue) and B recognition element (BRE) (CRnAAT, purple) (where W is A or T, R is A or G, and n is any nucleotide base). Panel A, the transposon insertion sites of isolates 7 and 35A identified 250 bp and 42 bp upstream of the start codon of *hvo_2469*, respectively. Panel B, transposon insertion site of isolate 36A identified 37 bp upstream of the start codon of *hvo_1957*.

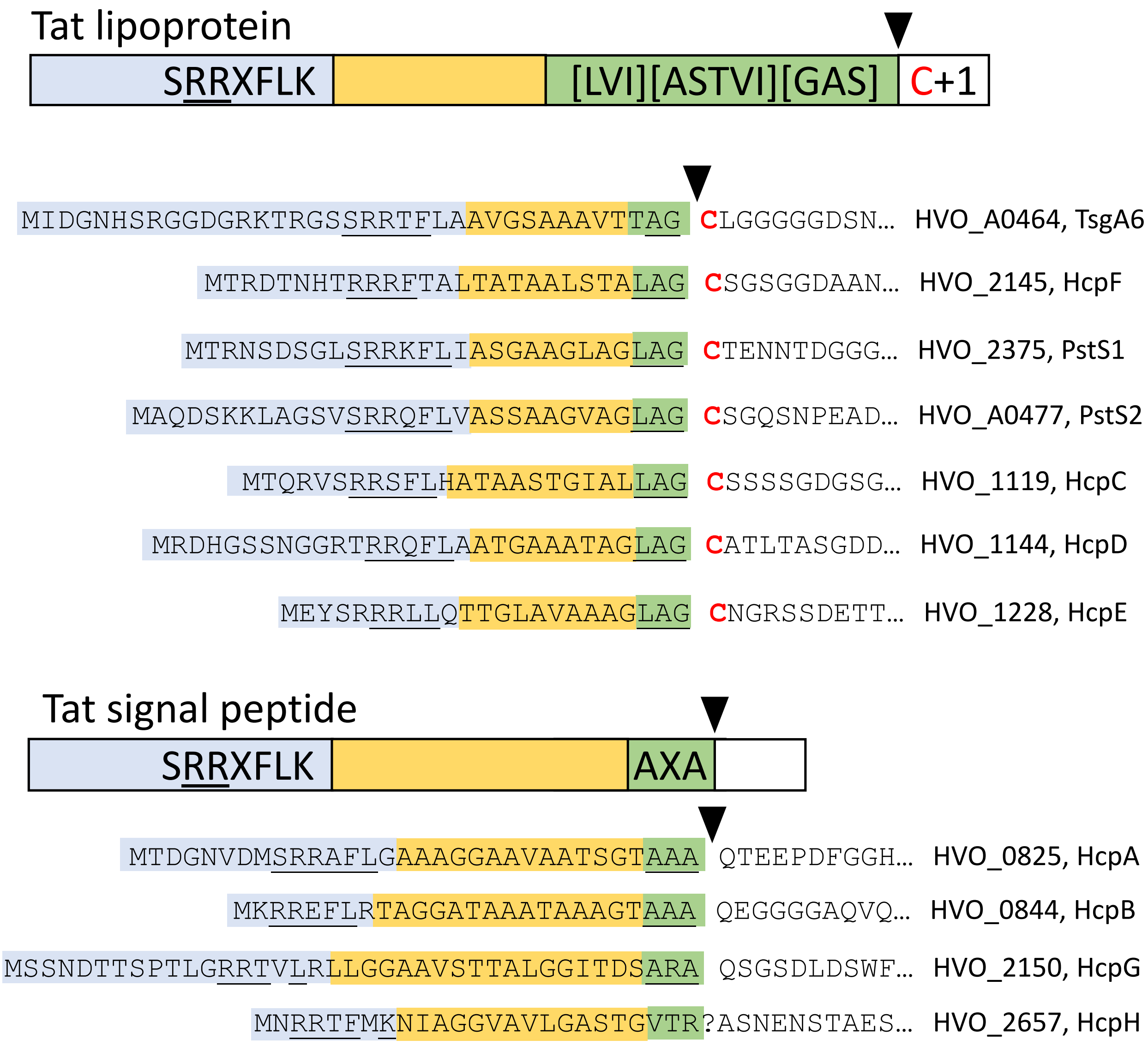

Figure S2. Twin arginine translocation **(**Tat) signal peptide and lipoprotein motifs of select *H. volcanii* proteins. Transposon insertions in the coding sequences of HVO_A0464 (TsgA6, solute binding protein), HVO_2145 (HycF, halocyanin) and HVO_2375 (PstS1, solute binding protein of Pi transport) render cells hypertolerant to hypochlorite stress (top three proteins listed). All three polypeptides are predicted to be translocated via the cell membrane by the Tat system and cleaved at the P2-P1-P1′ site of Ala-Gly-Cys consensus to generate a N-terminal cysteine residue (in red) that is lipid modified based on analogy to halocyanin [56]. Predicted site of cleavage is indicated by a down arrow. Other proteins discussed in the text are included for comparison. Blue, positively charged N-domain; yellow, hydrophobic H-domain; green, C-domain of the Tat lipoprotein and Tat signal peptides. The SRRXFLK, [LVI][ASTVI][GAS] and AXA consensus sequences are underlined in the protein sequence (where X represents any amino acid).

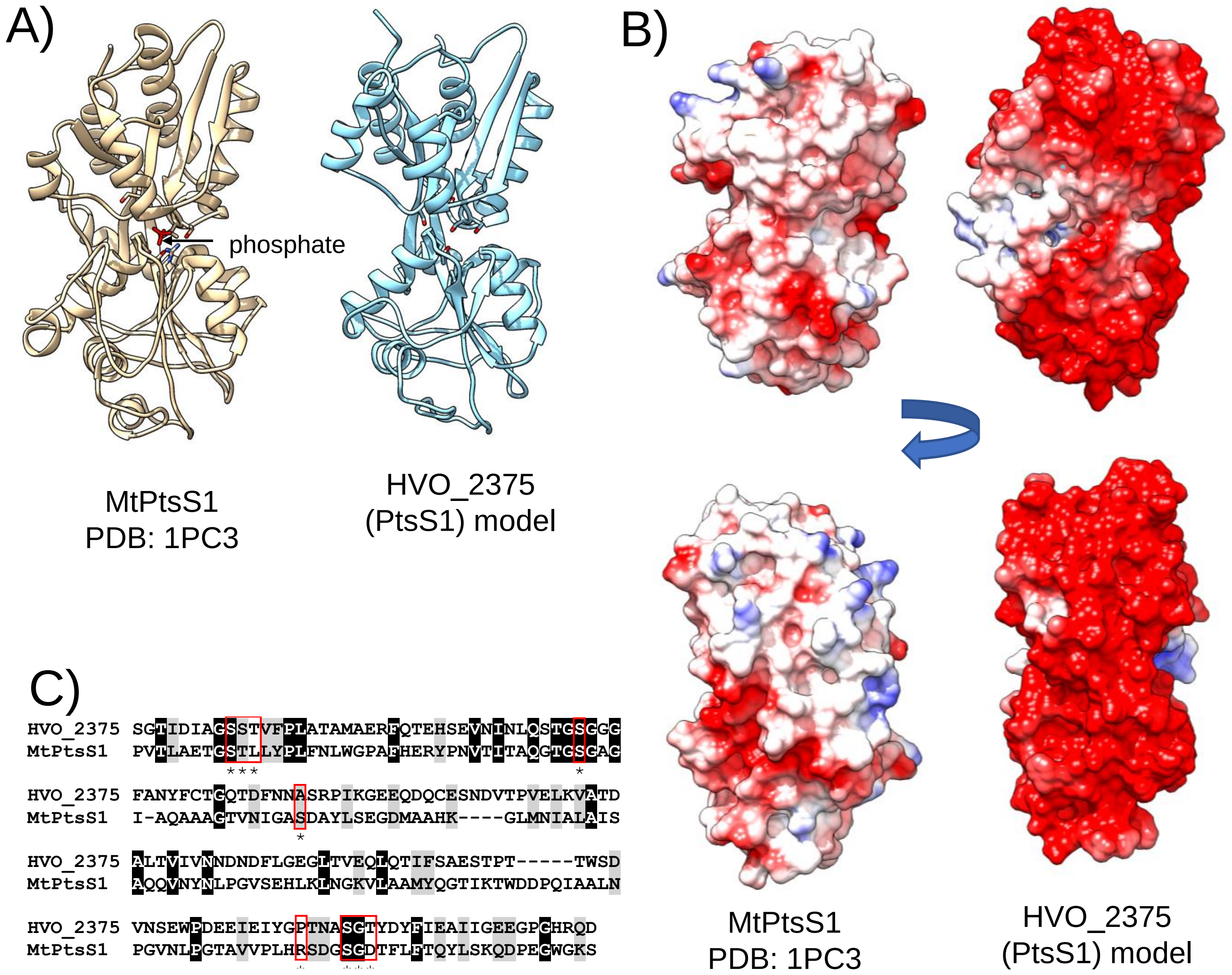

Figure S3. *H. volcanii* PstS1 (HVO_2375) is structurally related to bacterial phosphate (Pi) solute binding proteins. A-B) Phyre2-based structural homology model of HVO_2375 and its relationship at an RMSD of 1.057 Å to the X-ray crystal structure of the Pi solute binding protein *Mycobacterium tuberbulosis* MtPtsS1 (PDB: 1PC3). A) MtPtsS1 residues that coordinate Pi are conserved in HVO_2375 as represented by stick on ribbon diagram with the bound Pi indicated by an arrow and C) as red boxes/asterisk in a multiple amino acid sequence alignment. B) Surface representation of HVO_2375 and MtPtsS1 reveals both proteins completely engulf the bound Pi, but differ in surface charge. By contrast to MtPtsS1, HVO_2375 has a highly acidic shell which likely promotes the activity/stability of this protein in the hypersaline environments. The basic/neutral patch of HVO_2375 may promote interaction with partner proteins. Coulombic surface coloring is used to represent the electrostatic potential with units in the range of -10 (red), 0 (white), and 10 (blue) kcal/mol*e using Chimera v 1.7. The N-terminal Tat motif predicted for HVO_2375 is not included in the models or the multiple amino acid sequence alignment.

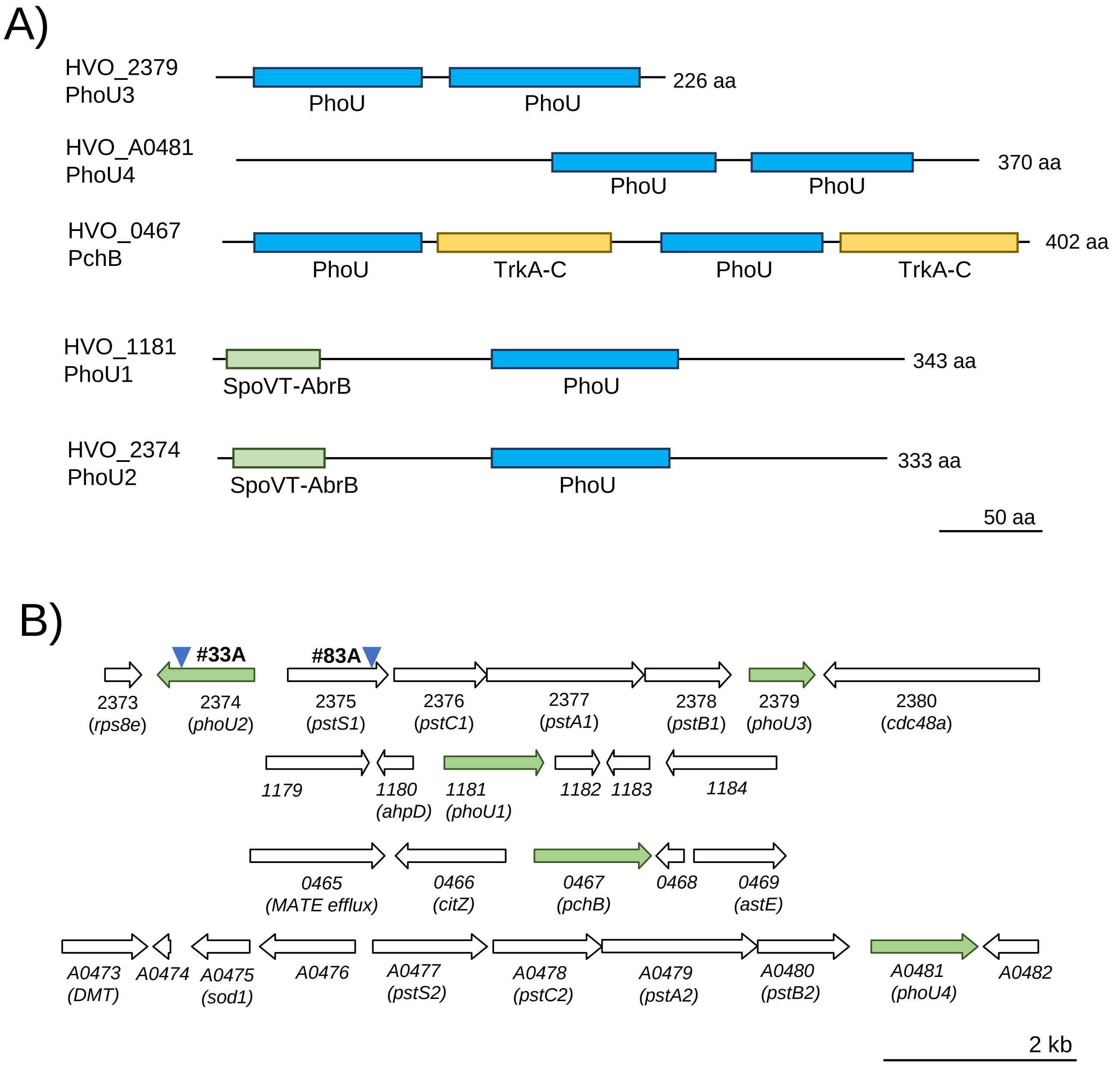

Figure S4. PhoU domain family proteins of *H. volcanii*. A) Protein domain architecture including the SpoVT-AbrB DNA binding domain, TrkA-C regulator of K^+ conductance (RCK) domain and PhoU domain. Scale bar, 50 aa. B) Genomic neighborhood of *phoU* related genes. Down arrowheads (dark blue) represent the site of transposon insertion with the strain number indicated adjacent to the symbol. Arrows (green or white) represent ORFs deduced from the DNA sequence of the *H. volcanii* genome. HVO_ locus tag number and gene names are indicated below the ORF. Genes encoding proteins with one or more PhoU domains are indicated in green. Scale bar, 2 kb.

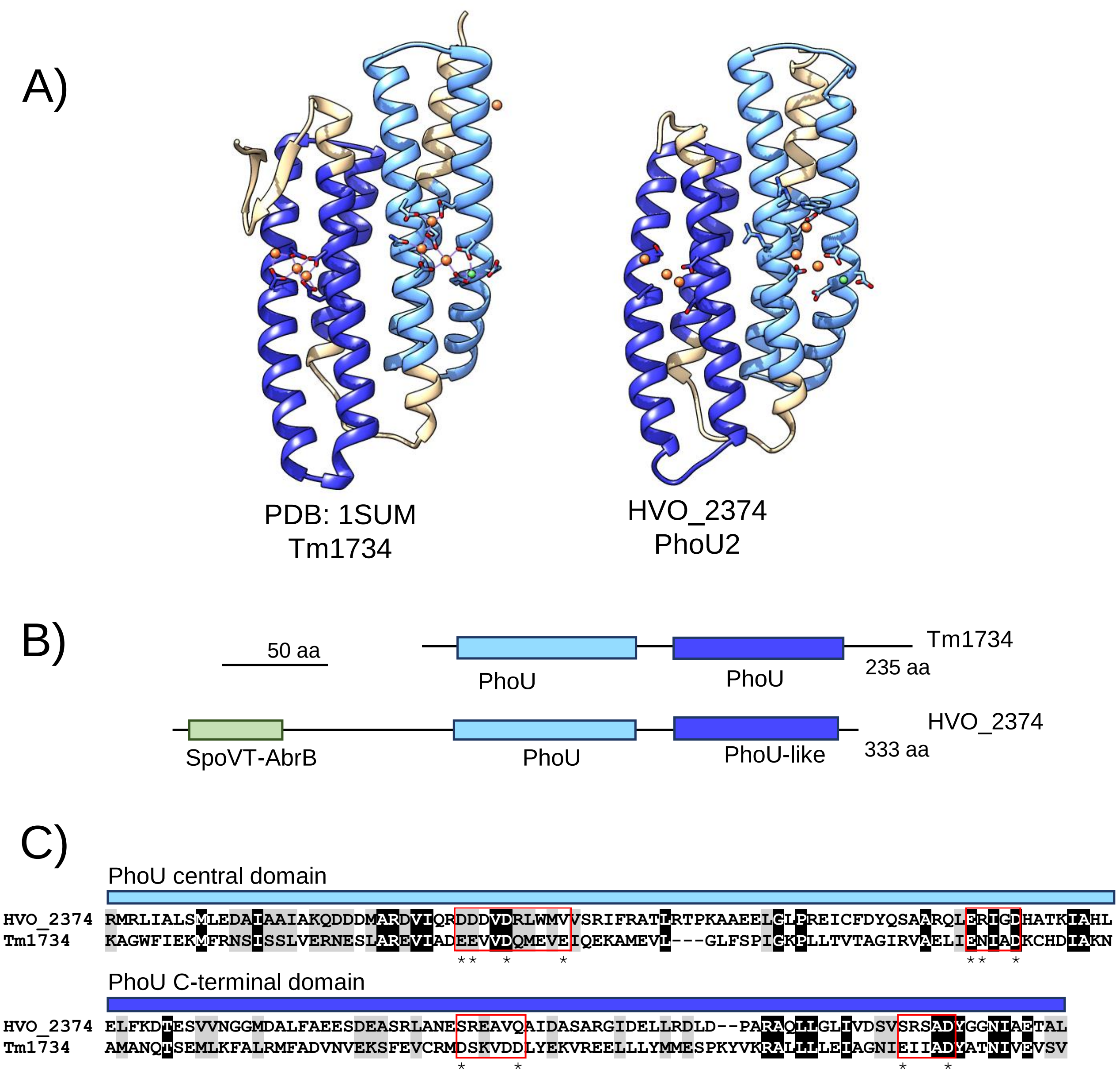

Figure S5. *H. volcanii* PhoU2 (HVO_2374) and its relationship to *Thermotoga maritima* Tm1734. A) Ribbon diagram of a Phyre2-based structural homology model of HVO_2374 compared to the X-ray crystal structure of Tm1734 (PDB: 1SUM) at RMSD of 0.724 Å. The tandem PhoU domains are highlighted in light and dark blue. Fe and Ni are represented by orange and green balls. B) Protein domain architecture with the PhoU domains colored in light and dark blue as in panel A. The N-terminal SpoVT-AbrB superfamily (IPR037914) DNA binding domain is indicated in green. Scale bar, 50 aa. C) Multiple amino acid sequence alignment of the PhoU domains colored as in panels A-B. The red boxes highlight the two multinuclear iron clusters coordinated by a conserved E(D)XXXD motif pair in the Tm1734 structure. Only the first of the two motifs is conserved in HVO_2374.

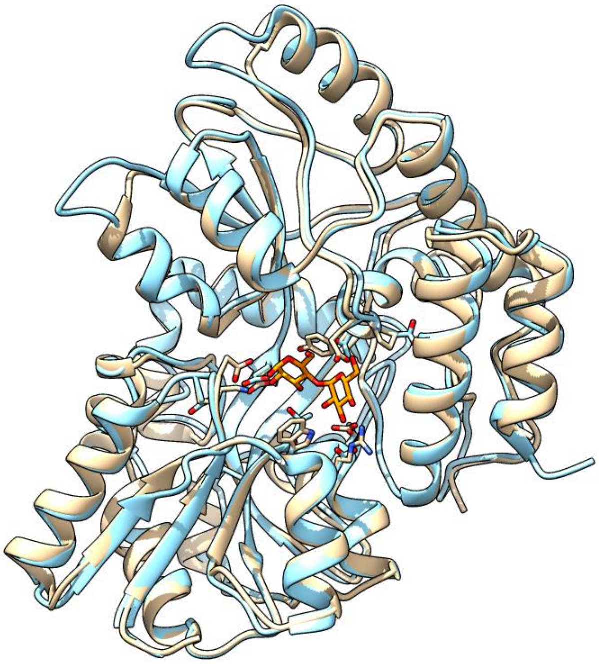

MalE	HVO_A0494
D14	G57
K15	D58
W62	D108
A63	T109
D65	T111
R66	D112
**E111**	**E152**
E153	A199
P154	D200
Y155	T201

Figure S6. Comparison of *H. volcanii* TsgA6 (HVO_A0494) to the *E. coli* maltose binding protein (MalE). The structural homology model of HVO_A0494 (blue ribbon) is based on Phyre2-comparison to the PDB database and is related to the X-ray crystal structure of the *E. coli* MalE (PDB: 3OSQ, tan ribbon) at an RMSD of 0.371 Å. Residues in the active site region of MalE are indicated by tan stick diagram and in the table inset which highlights the single residue of HVO_A0494 that is conserved in this region (colored in green). Maltose is in orange stick diagram.

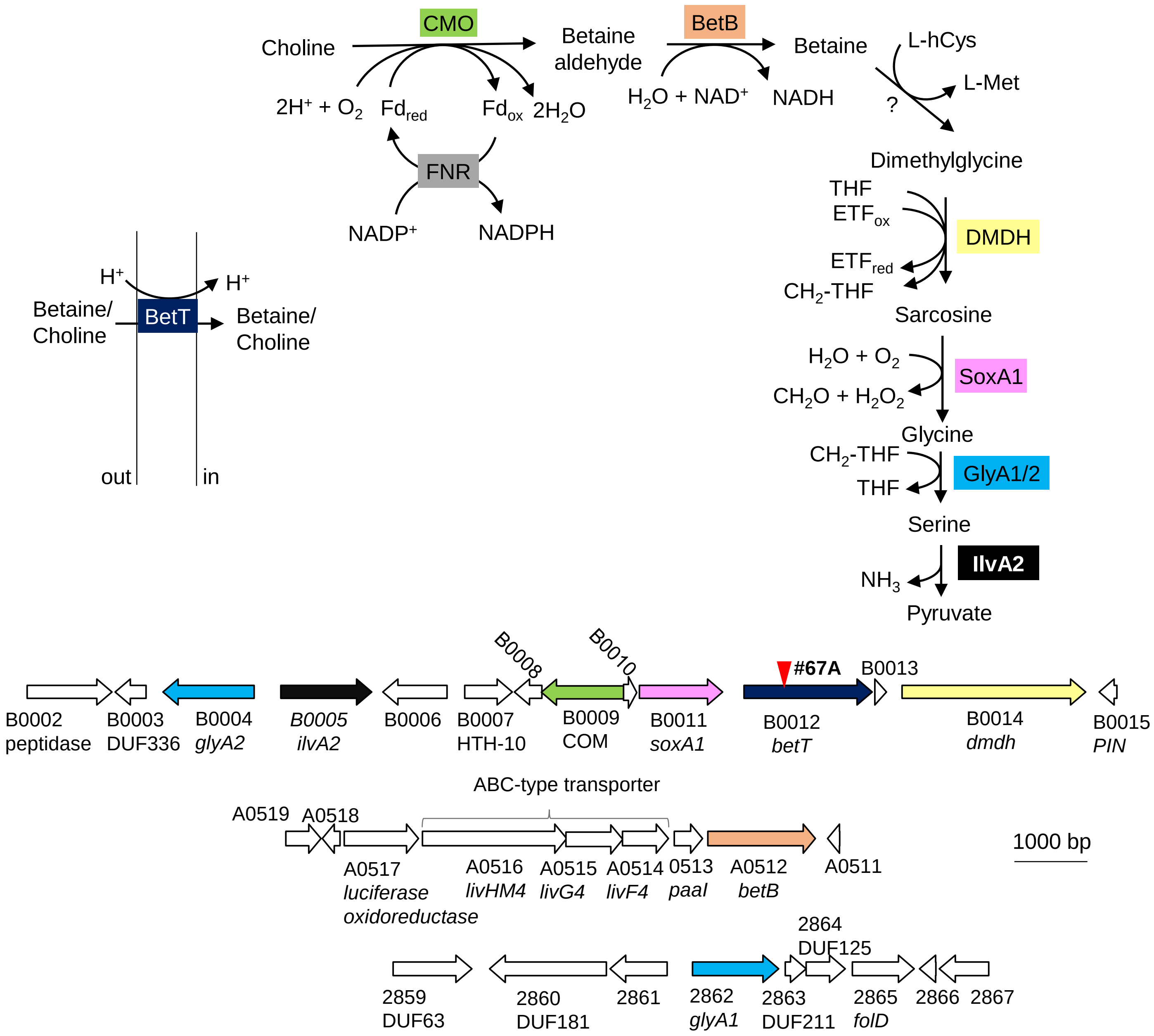

Figure S7. *H. volcanii* HVO_B0012 (BetT) and its predicted function in choline transport. The betaine/choline metabolic pathway of *H. volcanii* is based on comparison to bacteria and plants (KEGG pathway) [73]. Genes, represented as arrows, are colored in agreement with the encoded enzymes represented in the pathway. Abbreviations: CMO, choline monooxygenase [EC 1.14.15.7]; BetB, betaine aldehyde dehydrogenase [EC 1.2.1.8]; DMDH, dimethylglycine dehydrogenase [EC 1.5.8.4]; SoxA1, sarcosine oxidase [EC 1.5.3.1]; GlyA1/2, glycine hydroxymethyltransferase [EC 2.1.2.1]; IlvA2, L-serine/L-threonine ammonia-lyase [EC 4.3.1.17, 4.3.1.19]; ox, oxidized; red, reduced; L-Met, L-methionine; L-hCys, L-homocysteine; THF, tetrahydrofolate; CH_2-THF, N5,N10-methylenetetrahydrofolate; THF, tetrahydrofolate; FNR, ferredoxin-$NADP^+$ reductase [EC 1.18.1.2] ($F420H_2$: NAD(P)H oxidoreductase HVO_0433 and ferredoxin reductase-like HVO_0889); HVO_B0006, disordered regions include 105-132 aa and 180-215 aa (MobiDB_lite); HVO_B0003, homolog of HbpS regulator up-regulated in response to haemin- and peroxide-based oxidative stress [74]; HVO_B0008, structural homolog of the C-terminus of minichromosome2 maintenance (MCM) protein (PDB: 2MA3); Fd, ferredoxin (HVO_0211 FerB1, HVO_2729 FerB2, HVO_1246 FerA1, HVO_1482 FerA2, HVO_1721 FerA3, HVO_1831 FerA4, and HVO_2995 FerA5); ETF, electron transferring flavoprotein (HVO_0304 EtfA1, HVO_0305 EtfB1, HVO_2731 EtfA2, HVO_2730 EtfB2, HVO_2732 FixC).

A)

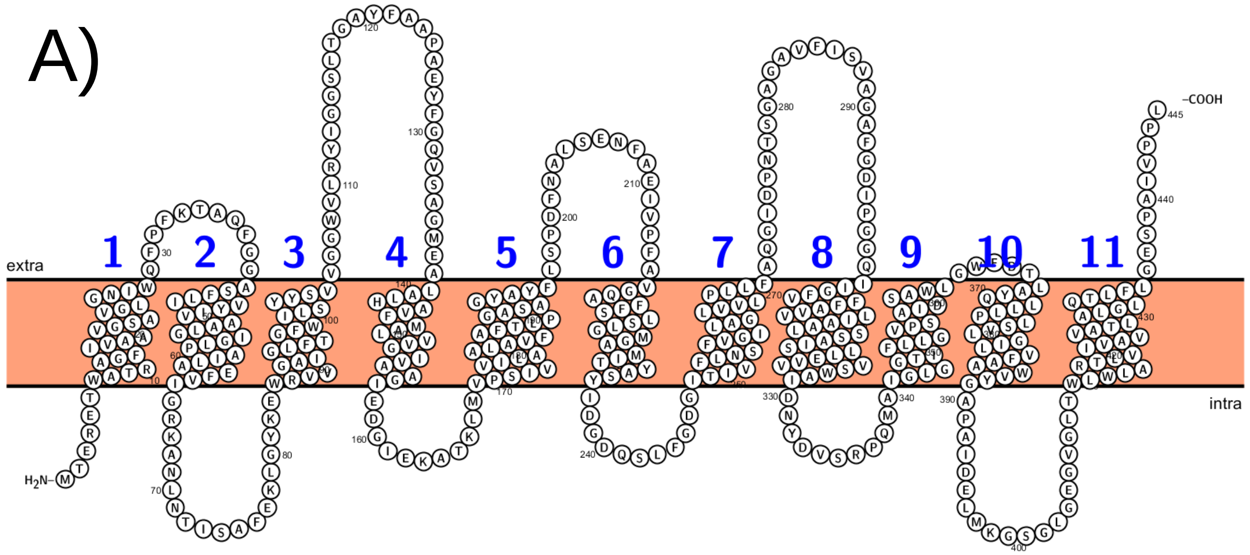

B)

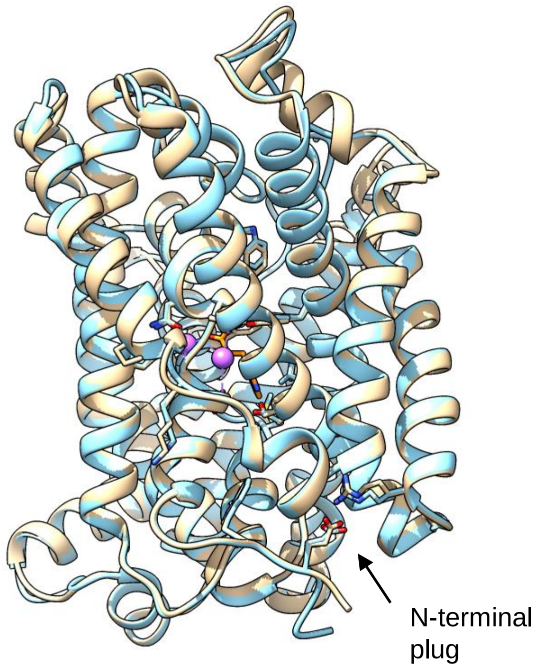

MhsT	HVO_2469
Trp33	Trp28
Tyr103	Tyr102
Ser233	Ser224
Gly30	Gly25
Ala26	Ala21
Thr231	Ser222
Phe230	Phe221
Met236	Met227
Ser327	Ser319
Leu328	Leu320
Asn31	Asn26
Asp263	Asn254
Trp12	Trp7
Arg344	Agr336
Glu10	Glu5
Gly171	Gly160
Pro181	Pro170
Lys177	Lys166
Gly28	Gly23
Ala320	Ala312

Figure S8. *H. volcanii* SNF family transporter homolog HVO_2469. Panel A) 2D transmembrane diagram of HVO_2469 generated using Protter [75]. Cell membrane is represented in orange. Panel B) 3D homology model of HVO_2469 (blue) compared to the X-ray crystal structure of the bacterial Na^+-dependent SNF family transporter MhsT (PDB: 4US3, tan) at a RMSD of 0.262 Å. MhsT residues that plug the cytoplasmic side and coordinate the two Na^+ ions (purple balls) and L-tryptophan (orange stick) are listed in the inset, with the conserved residues of HVO_2469 indicted in green.

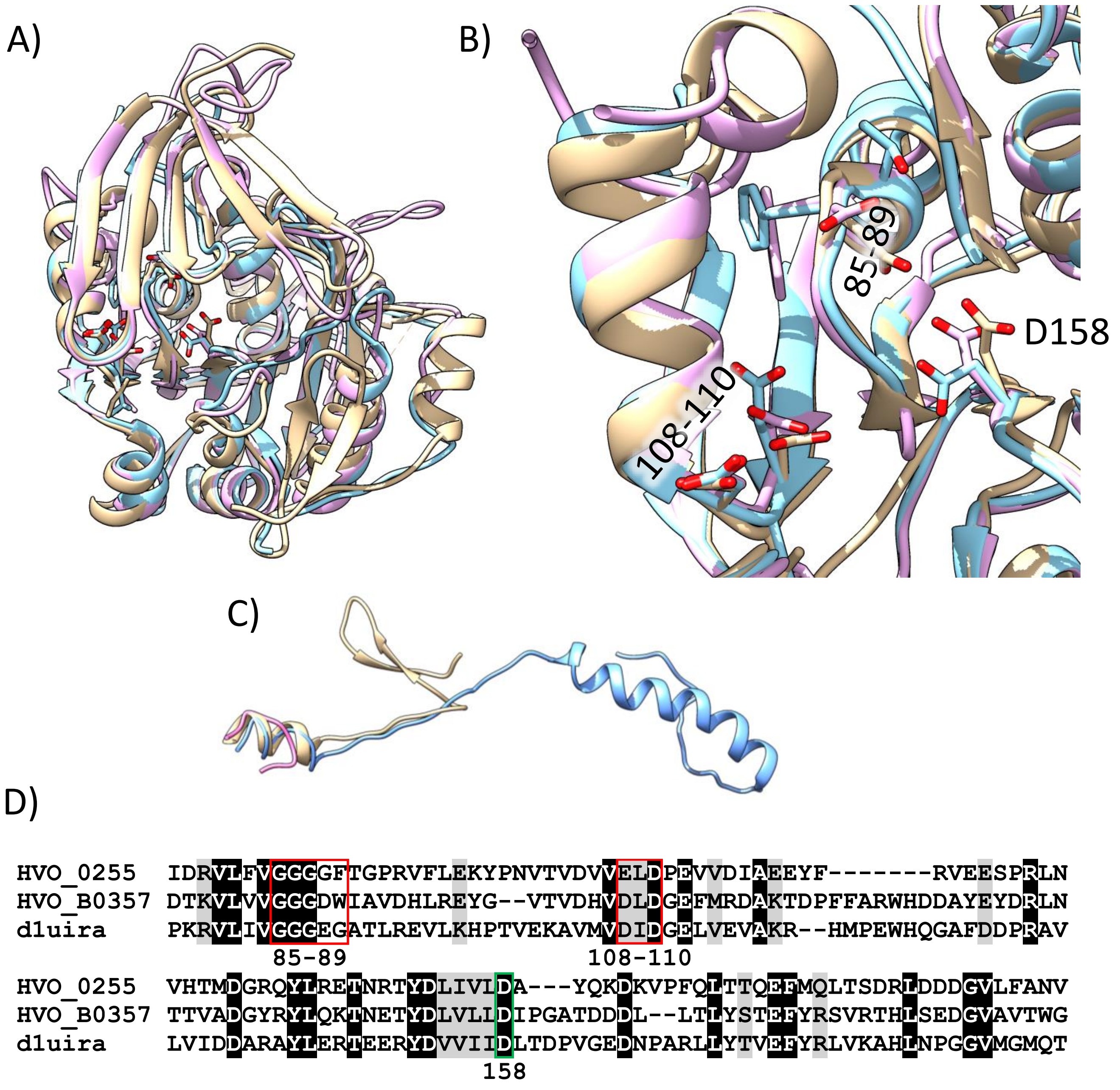

Figure S9. *H. volcanii* SpeE homologs related to the polyamine aminopropyltransferase of *Thermus thermophilus*. Panel A) 3D homology models of the soluble domain of the *H. volcanii* SpeE homologs HVO_0255 (blue) and HVO_B0357 (pink) compared to the X-ray crystal structure of *T. thermophilus* triamine/agmatine aminopropyltransferase (PDB: 1UIR, tan) at a RMSD of 0.905 and 0.804 Å, respectively. Panel B) Zoom-in of the conserved motifs described in panel D. Panel C) C-terminal extension of HVO_0255 and 1UIR, which is not apparent in HVO_B0357. Panel D) Zoom-in of the conserved GGG(D/E)G and (E/D)(I/V)D motifs important for binding polyamine and the amino donor S-adenosylmethioninamine (red boxes) and the proton acceptor of the reaction (green box). Residues are numbered according to 1UIR.

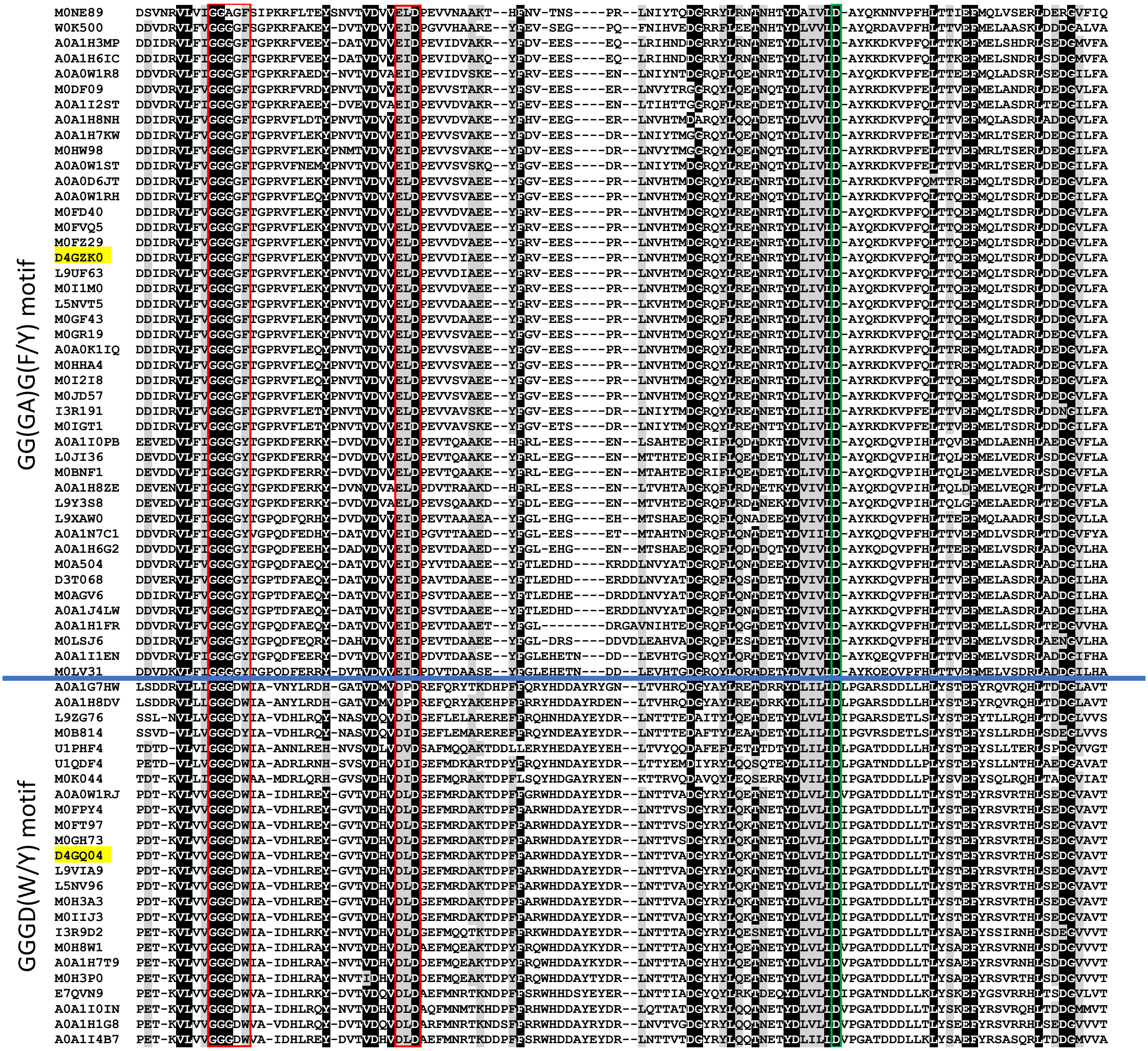

Figure S10. Multiple amino acid sequence alignment of the active site region of haloarchaeal members of the SpeE family. GGG(D/E)G and (E/D)(I/V)D motifs important for binding polyamine and the amino donor S-adenosylmethioninamine (red boxes) and the conserved proton acceptor of the reaction (green box) are highlighted. Uniprot number is on the left where HVO_B0357 (D4GQ04) and HVO_0255 (D4GZK0) are indicated in yellow.

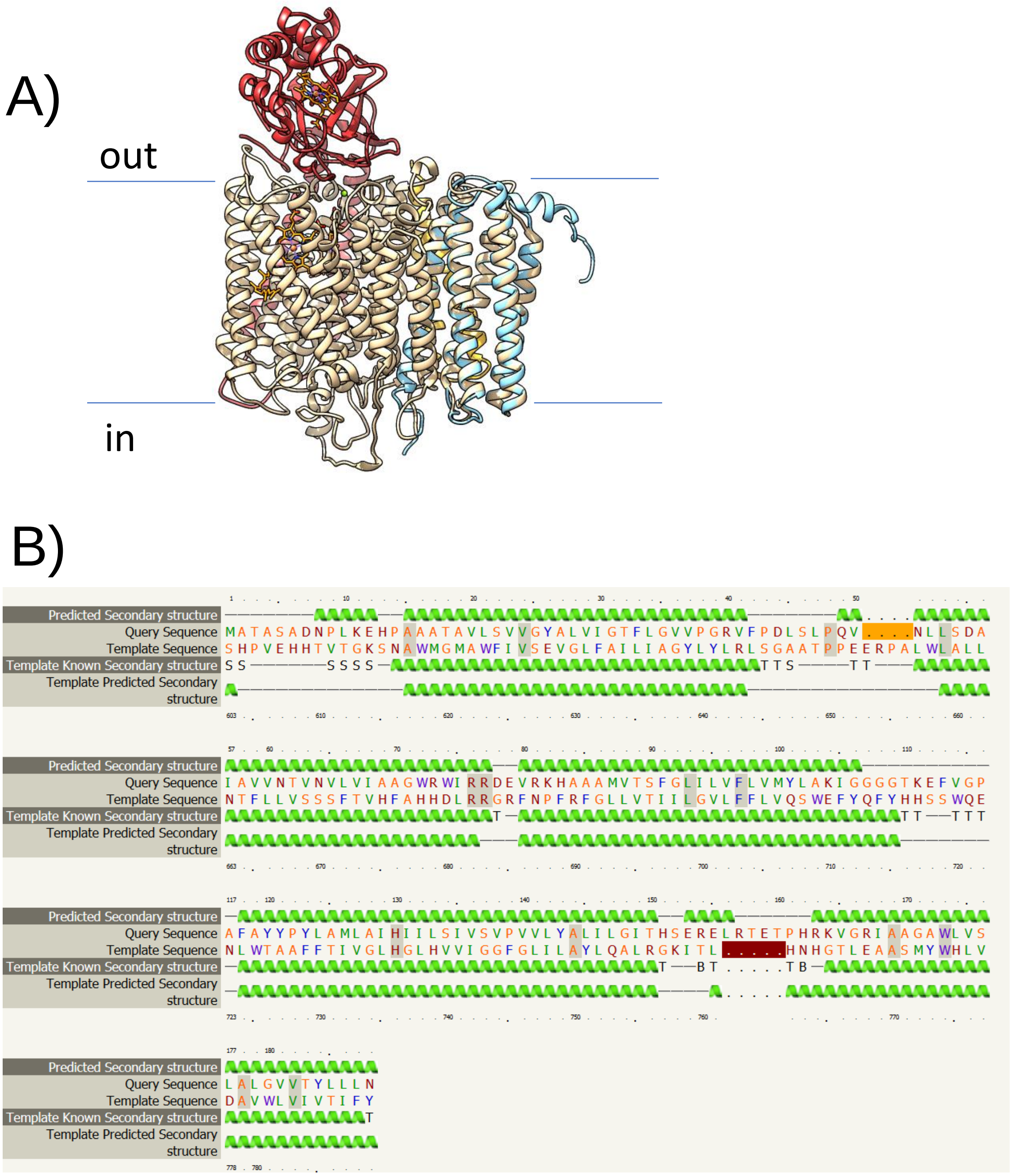

Figure S11. *H. volcanii* HVO_0823 is a cytochrome *c*-oxidase (EC: 1.9.3.1) type helical bundle protein. A) The 3D structure of HVO_0823 (blue) generated by Phyre2-modeling compared to the X-ray crystal structure of the *caa3*-type cytochrome oxidase of *Thermus thermophilus* (PDB: 2YEV, tan, golden and salmon) at an RMSD value of 0.672 Å. The heam sites are in orange stick. B) The predicted secondary structure and primary amino acid sequence alignment of HVO_0823 compared to the C-terminal domain of the C-terminal domain of the polypeptide I+III (CaaA) of the *caa3*-type cytochrome oxidase complex (PDB: 2YEV).

A)

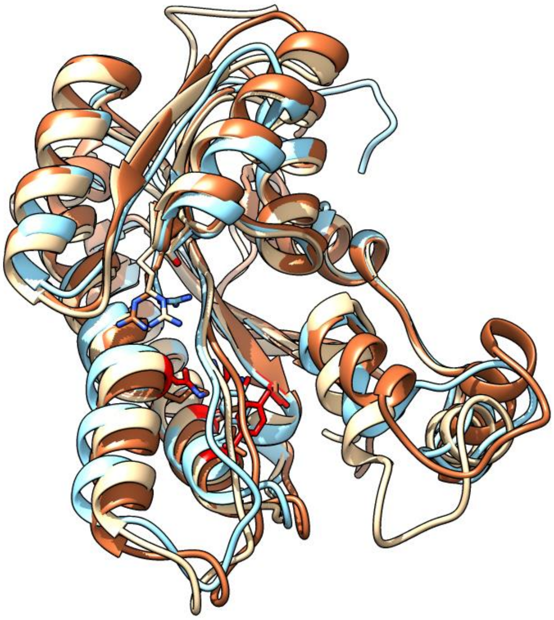

3LF2	1X7H	HVO_2504
Lys118	Asn114	Gly114
Ser146	Ser144	Gly142
Thr159	Tyr157	Tyr155
Arg163	Lys161	Lys159

B)

```
MG2_0210       M-FERFKKRSQARISMGTAVITGANRGLGAEWVSQLIEQGWTVYAGYRSDMGRACDALSTHQLDVQDRASVQAFAD---A
ZOD2009_16718  -----MNIDLYDSLDGQVALVTGANRGIGEEIATRLAEFGATVYAGARDPDAVTAPDQRAVRLDVTEEETIRGAVGTIAD
HQ2380A        -----MKVETYDSLSGQVALVTGANRGIGAEIARMLAEEGATVYAGARRPETVTASNQRAIEIDVTDDTQMESAISTIDD
rrnAC3483      -----MDVPLYDSLDSQVVLVTGATRGIGKAIADGLVDLDATVYAGARDTDDIEATDRHAIELDVTDDDGMVAAVDRIER
HAH_0704       -----MDVPLYDSLDGQIVLVTGATRGIGKAIADGLVDLDATVYAGARDTGDIAATDRHAIELDVTDDDGMVAAVDRIER
Huta_2460      -----MEPNLLETLDGQVALVTGANRGIGREIASGLVELDATVYGGVRDPTAALPDGVEAVEIDVTEESMIESAVEEVVS
HLRTI_01047    -----MEPNLLETLDGQVALVTGANRGIGREIASGLVELDATVYGGVRDPTAALPDGVEAVEIDVTDESMIESAVEEVVT
Hbor_09100     MTADVPDPDLYDSLEGQVALVTGANRGLGREIAEQLHDLGATVFAATRSITHDIPDEWNHLLVDVTQEGEISDAVDDIFA
HQ2771A        MSTALSEPSLYESLDGQVAVVTGANRGLGRQIAENLDELGATVFTATRSMTHDIPDSWEHLLVDVTQSGEIADAADEIFS
HVO_2504       -----MEPDRYDDLDGQVALVTGANRGIGRQIAANLRDRGATVYAGSRSVTNETPEETERVLLDVTQEGDIEEVVDGIFA
                                    17/18 22 24                43

MG2_0210       APAKIDLLVNNAGVADGRRNISEIDDVWALEVIDINALGPVRVVQALYGKMNHNSLTKVAMVSSLMASIDDCHMGRSYAY
ZOD2009_16718  EAGRLDVLVNNAATYGPTGKLDSLDSETIETTLRTNLHGPMLVTKHALSLLTVRDGGRVVTLSSGSGQFDGGIDTGHLPY
HQ2380A        AEGQLDILVNNAGVRGPTTSLEDAPIDEIDQTFAVNLRGPVVLTKYALPLLREQAGARVVNLSSGLGAIGPGMDGGSPPY
rrnAC3483      EQGRLDVLVNNAGVMDSREPLDGMPTDVIDHTLDTNLRGAVLMTKYALPLLLAEAGGRIVAMSSGLGAITESQSGGTPAY
HAH_0704       EQGRLDVLVNNAGVMDSREPLDEMPTDVIDHTLDTNLRGAVLMTKYALPLLLAEEGGRVVTMASGLGAITESQSGGTPAY
Huta_2460      AGGQLDILVNNAGVGGAGAGIEDIDSEDFDRILDVNLRGPSLLSKYALPHLLETDAPRIVNVSSGVGILAEPIESEMPAY
HLRTI_01047    AGGQLDVLVNNAGVGGAGAGLTDVDSEAFDRILDVNLRGPSLLSKYALPHLLETDAPRIVNVSSGVGILADPIESEMPAY
Hbor_09100     SAGRLDILVNNAGISGGDGDIVAESVTDIDQTLATNLRGPMLVCKHAVPLLVQDEGGRVINVSSGMGALNEAQSGGSPSY
HQ2771A        IAGRLDIVVNNAGIGGGDGDIVSESIDDINRTLSTNLRGPMLVCKHTVPLLLQNDGGRIVNVGSALGMLSREQSGGNPAY
HVO_2504       DQNRLDVLVNNAGVGGEGEDIVAEPTERIDRTLGVNLRGPMLLCKHAVPLLLQSDGGRVVNVSSGMGALGEEQSGGSPAY
                          86                                                   139            155

MG2_0210       RASKTALNMFTVSMKKEAIEDQISFVILHPGWVKTSMGGDRAPVEIPDSVKGMRNVVDALTLETSGRFIFDGELLPW
ZOD2009_16718  GVSKAGVNAFTDALSTQYPN--LLVNAVCPGWVRTDMGGSGAPRSVEKGAETPVWLARFEPGNPSGKLWRDRSVIEW
HQ2380A        RVSKTGLNGFTAYLHGEYATEGLISNAARPGWVQTELATPGAPRTPTEGADTPVWLARFKPNAPAGRCWRDRMPIGW
rrnAC3483      RISKTGVNGLTKYLDGEYAADGLIANSVCPGYVQTDMTEGSAPRTPEKGAETPVWLARFQPDAPSGRFWRDKAEIEW
HAH_0704       RISKTGVNGLTKYLDGEYAADGLIANSVCPGYVQTDMTEGSAPRTPEKGAETPVWLARFRPDAPSGRFWRDRAEIEW
Huta_2460      RISKAGINALTVSLDQTYGEDGLIANSVDPGWVATDLGGEEAPREPQKGAETPIWLSRFGPGSPSGLFWKDKQVIDF
HLRTI_01047    RISKAGINALTVSLDQTHGEDGLIANSVDPGWVATDLGGEEAPREPEKGAETPIWLSRFGPGSPSGLFWKDKQVIDF
Hbor_09100     RISKTGLNGLTKYLDGEYGDDGLLANSVCPGWVRTDMGGEDADRSVERGAETPVWLCRFKPESPSGYFWRDQSVIDW
HQ2771A        RVSKSGLNGLTAYLDGEYGSDGLIANSVCPGWVRTDMGGPDADRSIKTGAETPTWLCRFRPESPRGHFWRDRAVIEW
HVO_2504       RISKTGLNGLTTYLDGQYGDDGLIANSVCPGWVRTDMGGEEANRPVEKGAETPTWLATFKPGAPAGKFWRDKEVIDW
                 159    166            182
```

Figure. S12. HVO_2504 is a putative oxidoreductase of the short-chain dehydrogenase/reductase (SDR, IPR002347) family. Panel A) 3D homology model of HVO_2504 (blue ribbon) overlaid on the X-ray crystal structures of an actinorhodin polyketide ketoreductase (PDB: 1X7H, tan) and a putative short chain oxidoreductase (Q9HYA2, PDB: 3LF2, brown). The catalytic tetrad Asn114-Ser144-Tyr157-Lys161 of the ketoreductase is highlighted in red in the structure and is compared to the atypical residues of HVO_2504 and Q9HYA2, with residues conserved with the tetrad indicated in green in the table inset. Panel B) Multiple amino acid sequence alignment of arCOG01266 members with conserved residues of the TGxxxGxG motif or within the central cavity of the HVO_2504 3D-model highlighted in red. Other conserved residues are in black/grey.

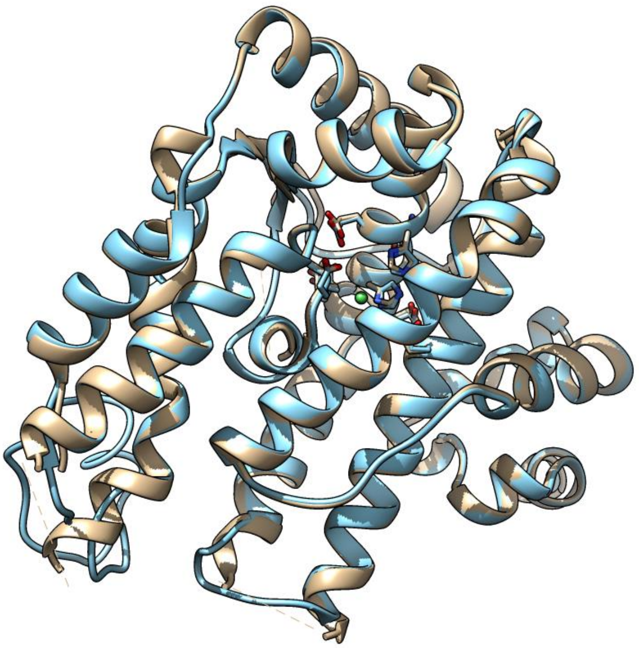

3CMN	HVO_2770
Tyr168	Tyr132
Asp169	Asp133
**His210**	**His174**
**Glu211**	**Glu175**
**His214**	**His178**
Glu217	Glu181
Glu294	Glu224

Figure S13. DUF2342 protein HVO_2770 is related to zinicin-like metallopeptidase type 2 (IPR018766) and F420 biosynthesis associated (IPR022454) families. The 3D structure of HVO_2770 (blue) generated by Phyre2-modeling against the PDB database was compared to the x-ray crystal structure of a putative hydrolase with a novel fold from *Chloroflexus aurantiacus* (PDB: 3CMN, tan) at an RMSD value of 0.354 Å. Conserved residues of a putative metal-binding active site including the HExH motif (red) and other residues (green) are indicated in the table inset.

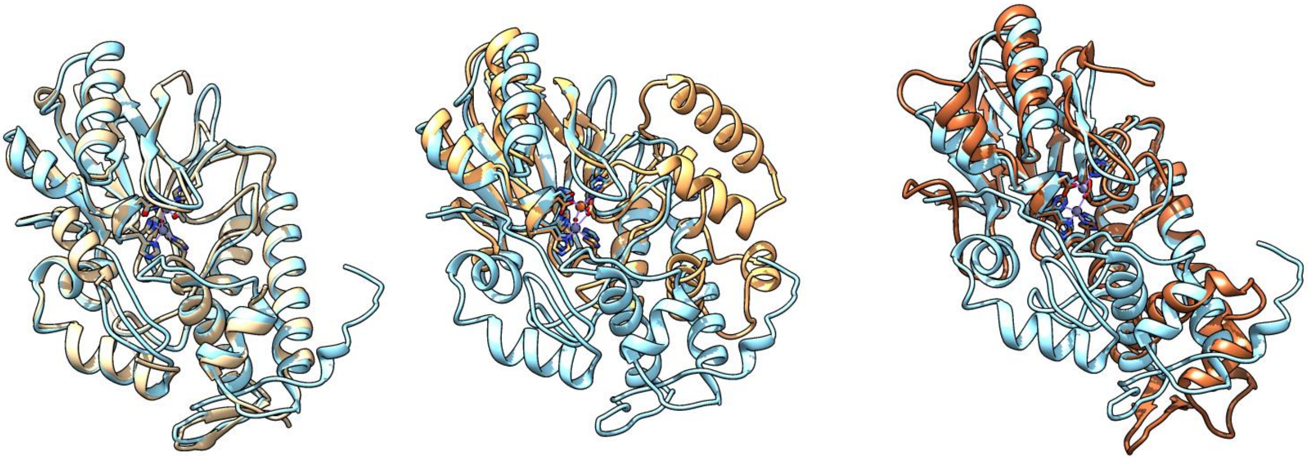

PDB: 2ZO4 PDB: 1XM8 PDB: 4AD9

Metal site	2ZO4	HVO_1041
1	His70	**His62**
1	His72	**His64**
1	His171	**His164**
2	Asp74	**Asp66**
2	His75	**His67**
2	Asp190	**Asp187**
2	His233	**His230**

Figure S14. *H. volcanii* HVO_1041 is a metallo-β-lactamase superfamily protein. The 3D structure of HVO_0823 (blue) generated by Phyre2-modeling against the PDB database was compared to the x-ray crystal structures of related metallo-β-lactamase superfamily proteins including from left to right: (i) TTHA1429 of unknown function from *Thermus thermophilus* HB8 (PDB: 2ZO4, tan) at an RMSD value of 0.589 Å, (ii) a mitochondrial glyoxalase II from *Arabidopsis thaliana* (PDB: 1XM8) at an RMSD value of 1.085 Å, and (iii) a human mitochondrial endoribonuclease (PDB: 4AD9) at an RMSD value of 0.931 Å. Residues that coordinate the two Zn^{2+} ions of TTHA1429 and the analogous residues of HVO_1041 are indicated in green in the table inset.

Table S1. Strains, plasmids, and oligonucleotide primers used in this study[a].

Strain, plasmid, or primer	Description[a]	Source or Ref.
E. coli strains:		
TOP10	F⁻ *recA1 endA1 hsdR17*(r_K^- m_K^+) *supE44 thi-1gyrA relA1*	Invitrogen
GM2163	F⁻ *ara-14 leuB6 fhuA31 lacY1 tsx78 glnV44 galK2 galT22 mcrA dcm-6 hisG4 rfbD1 rpsL136 dam13*::Tn*9 xylA5 mtl-1 thi-1 mcrB1 hsdR2*	New England Biolabs
H. volcanii strains:		
H295	*ΔpyrE2 bgaHa-Kp ΔtrpA Δmre11 Δrad50*	[76]
Transposon library	H295 with random Mu-like transposon insertions	[11]
DS70	wild-type isolate DS2 cured of plasmid pHV2	[77]
H26	DS70 *ΔpyrE2*	[78]
HM1041	H26 *Δsamp1*	[79]
HM1052	H26 *ΔubaA*	[79]
GZ130	H26 *ΔpsmA1*	[72]
GZ114	H26 *ΔpsmC (= psmA2)*	[72]
GZ109	H26 *ΔpanA/1*	[72]
GZ108	H26 *ΔpanB/2*	[72]
Plasmids:		
pTA131	Ap[r]; pBluescript II containing P_{fdx}-pyrE2	[78]
Primers:		
M1-1F (TL Inverse Forward)	5′-cctcgacctcgtgttcatcgtcgcgccgacgacc-3′	This study
M1-1R (TL Inverse Reverse)	5′-caaatattatacgcaaggcgacaaggt-3′	This study
M1-2F (TL Nested Forward)	5′-tcgaccgaatcatggaacaggtct-3′	This study
M1-2R (TL Nested Reverse)	5′-tacaacagtactgcgatgagtggc-3′	This study
M2-1F (HvTL_map_1st)	5′-atccggaacgcacataactgg-3′	This study
M2-1R (HvTL_map_degen)	5′-ctcggcattcctgctgaaccgctcttccgatctnnnnnnnnnntgacg-3′	This study
M2-2F (HvTL_map_2nd)	5′-gcatttatcgtgaaacgctttcg-3′	This study
M2_2R (HvTL_map_nested)	5′-tcctgctgaaccgctcttcc-3′	This study
C-HVO_0255_p	5′-ggcacgtcttcgactacacgtc-3′	This study
C-HVO_0823_p	5′-gaccgtctcgtggtcgtaccg-3′	This study
C-HVO_1003_p	5′-agacgagcttccagttcgagacgttcatcga-3′	This study
C-HVO_1091_p	5′-ttccatcagcggcgagcgagagcgcttgt-3′	This study
C-HVO_1092_p	5′-acctgaccgttctcgggttcgactacgccga-3′	This study
C-HVO_1956_p	5′-tccgagagaatcttttccgtgagcgt-3′	This study
C-HVO_2194_p	5′-cgtatcgctttttcgcggctgg-3′	This study
C-HVO_2469_p	5′-cgtccgtctactcaccgaaacca-3′	This study
C-HVO_2374_p	5′-ggtgacgtcgagactcatcgctt-3′	This study
C-HVO_2504_p	5′-agcggtcagctgttcgacatcgagacgctcaccga-3′	This study
C-HVO_2653_p	5′-tacgacaacttcagcaagaccgag-3′	This study
C-HVO_2770_p	5′-ggcacgtcttcgactacacgtc-3′	This study
C-HVO_C0005_p	5′-ttcgagaaccgcgaactgcccgacttta-3′	This study
Hvo_1957 RT-fw	5′-ggaactcgacgactactcc-3′	This study
Hvo_1957 RT-rv	5′-cagtactggtagtccgtgaa-3′	This study
Hvo_2469-RT-fw	5′-tgattacctacgcctcctac-3′	This study
Hvo_2469 RT-rv	5′-aagctgttcaggaagacgat-3′	This study

n, denotes any nucleotide in the degenerate primer. *H. volcanii pyrE2* (orotate phosphoribosyltransferase, HVO_0333), *trpA* (tryptophan synthetase A, HVO_0789), *mre11* (double stranded break repair protein, HVO_0853), *samp2* (ubiquitin-like SAMP2, HVO_0202), *ubaA* (E1, HVO_0558), *psmA* (or *psmA1*, α1 of 20S proteasome, HVO_1091), *psmC* (or *psmC1*, α2 of 20S proteasome, HVO_2923), *panA/1* (PanA/1 proteasome-associated AAA ATPase, HVO_0850), and *panB/2* (PanB/2 proteasome-associated AAA ATPase, HVO_1957).

Supplemental Table S2. Cycling conditions for the inverse-nested two-step PCR (INT-PCR) and semi-random two-step PCR (ST-PCR).

PCR step	Initial Denaturation	Amplification cycle	Final extension
INT-PCR<br>step 1 and step 2	98 °C, 30 sec	35 cycles of:<br>denaturation (98 °C, 10 sec) annealing (65 °C, 30 sec) elongation (72 °C, 2 min)	72 °C (5 min)
ST-PCR step 1	94 °C, 5 min	5 cycles of:<br>denaturation (94 °C, 30 sec) annealing (42 or 50 °C, 30 sec) elongation (72 °C, 3 min)<br>25 cycles of:<br>denaturation (94 °C, 30 sec) annealing (55 °C, 30 sec) elongation (72 °C, 3 min)	72 °C (5 min)
ST-PCR step 2	94 °C, 1 min	30 cycles of:<br>denaturation (94 °C, 30 sec) annealing (55 °C, 30 sec) elongation (72 °C, 3 min)	72 °C (5 min)

*All reactions were preheated to the initial denaturation temperature immediately prior to PCR.

Table S3. Identification of transposon insertion sites on the genome of *H. volcanii* mutant strains found hypertolerant of hypochlorite stress.

**Locus tag no. (gene)**	**Description**	**Isolate no.**	**Site of transposon**	**General method**	**General function**	**Predicted location**
HVO_0255 (*speE*)	Spermidine synthase (EC:2.5.1.16) of COG4262 super family which includes HVO_B0357	15A	Intra 320 bp before stop	INT-PCR (BmtI to BsphI)	Spermidine biosynthesis from putrescine (step 1)	Membrane (7 TM helices)
HVO_0823	DUF420 family, multipass TM protein with cytochrome c oxidase (EC:1.9.3.1) type helical bundle; Phyre2-based model to PDB: 1QLE	37A	Intra 45 bp before stop	INT-PCR (NdeI to NheI)	Electron transfer associated with the cell membrane	Membrane (5 TM helices)
HVO_1003 (*gufA2*)	ZIP family transport protein	57	Intra 22 bp after start	INT-PCR (HindIII to NdeI)	Metal ion transport	Membrane (7 TM helices)
HVO_1041	Metallo-beta-lactamase (IPR001279) domain protein	38A	Intra 47 bp after start	ST-PCR (42 or 50°C)	Metal binding hydrolase/ oxidoreductase	soluble
HVO_1091 (*psmA1*)	Proteasome subunit $\alpha 1$	59	Intra 5 bp after start	INT-PCR (HindIII to NdeI)	20S proteasome subunit	soluble
HVO_1957 (*panB2*)	Proteasome activating nucleotidase B2	36A	Inter 37 bp before start	INT-PCR (NdeI to NheI)	AAA ATPase associated with proteasome	soluble
HVO_2145 (*hcpF*)	Halocyanin blue (type I) copper redox proteins	65A	Intra 49 bp after start	ST-PCR (50°C)	Mobile electron carrier	Membrane associated (Tat lipoprotein)
HVO_2194 (*sir2*)	Sir2-type NAD-dependent protein deacetylase	16A	Intra 260 bp after start	INT-PCR (BmtI to BsphI)	Deacetylation of acetylated lysine residues of proteins	soluble
HVO_2374 (*phoU2*)	N-terminal SpoVT-AbrB DNA binding domain (IPR007159) and PhoU domain (IPR026022)	33A	Intra 221 bp before stop	INT-PCR (NdeI to NheI)	Regulation of phosphate uptake	Membrane associated (PhoU2, regulator of Pi transport; PstS1, Tat lipoprotein)
HVO_2375 (*pstS1*)	ABC-type transport system periplasmic substrate-binding protein (Probable substrate phosphate)	83A	Intra 169 bp before stop	ST-PCR (50°C)	Solute binding protein associated with phosphate transport	

HVO_2441 (*dppB5*)	ABC-type transport system permease DppB homolog	41A	Intra 503 bp after start	ST-PCR (42°C)	Transporter (permease) protein	Membrane (5 TM helices)
HVO_2469	SNF family transport protein; Phyre2-based 3D model	35A, 7	Inter before start by 42 bp for isolate 35A; Inter before start by 250 bp for isolate 7	INT-PCR (NdeI to NheI for isolate 35A and XhoI to BclI for isolate 7)	Na^+-coupled transporter (e.g., Na^+-dependent amino acid uptake)	Membrane (11 TM helices)
HVO_2504	Putative oxidoreductase (Short-chain dehydrogenase family)	62	Intra 23 bp before stop	INT-PCR (HindIII to NdeI)	NAD- or NADP-dependent oxidoreductase	soluble
HVO_2653	Uncharacterized protein (not amenable to Phyre2-3D modeling 01/16/18)	31A	Intra 105 bp after start	INT-PCR (NdeI to NheI)	unknown	Membrane (3 TM helices)
HVO_2770	DUF2342 protein of the zinicin-like metallopeptidase type 2 (IPR018766), and F420 biosynthesis associated (IPR022454) families	40, 30	Intra 140 bp after start for isolate 40; 142 bp after start for isolate 30	INT-PCR (HindIII to NdeI)	Metallohydrolase	soluble
HVO_A0494 (*tsgA6*)	TsgA6, ABC-type transport system periplasmic substrate-binding protein (probable substrate sugar)	63A	Intra 577 bp before stop	ST-PCR (42 or 50°C)	Transport (solute binding) protein	Membrane associated (Tat lipoprotein)
HVO_B0012 (*betT*)	Compatible solute transport protein (Probable substrate choline/glycine betaine)	67A	Intra 591 bp after start	ST-PCR (42 or 50°C)	Transporter (choline/glycine betaine)	Membrane (12 TM helices)
HVO_C0005	N-acylglucosamine 2-epimerase (EC 5.1.3.8) orthologous group POG093W01SY	93	Intra 93 bp before stop	INT-PCR (HindIII to NdeI)	Metabolism of carbohydrates associated with glycosylation of proteins and/or lipids	soluble

TM, transmembrane; Tn, transposon, NheI was a high fidelity (HF) enzyme; Intra, intragenic or within the open reading frame; Inter, intergenic; stop, stop codon; start, translational start site. Method 1, inverse-nested two-step PCR (INT-PCR) with the restriction enzymes used prior to ligation in parenthesis. Method 2, semi-random two-step PCR (ST-PCR) approach with the annealing temperature indicated in parenthesis.
